# Thiolated-Polymer-Based Nanoparticles as an Avant-Garde Approach for Anticancer Therapies—Reviewing Thiomers from Chitosan and Hyaluronic Acid

**DOI:** 10.3390/pharmaceutics13060854

**Published:** 2021-06-08

**Authors:** Roberto Grosso, M.-Violante de-Paz

**Affiliations:** Departamento Química Orgánica y Farmacéutica, Facultad de Farmacia, Universidad de Sevilla, 41012 Sevilla, Spain; robgrobul@alum.us.es

**Keywords:** thiomers, thiolated polymers, chitosan, hyaluronic acid, anticancer therapy, nanoformulations, nanotechnology, siRNA, taxanes, thiolation, preactivated, EPR effect, mucoadhesion, glutathione, smart DDS, permeation enhancer, disulfide bonds

## Abstract

Thiomers (or thiolated polymers) have broken through as avant-garde approaches in anticancer therapy. Their distinguished reactivity and properties, closely linked to their final applications, justify the extensive research conducted on their preparation and use as smart drug-delivery systems (DDSs). Multiple studies have demonstrated that thiomer-rich nanoformulations can overcome major drawbacks found when administering diverse active pharmaceutical ingredients (APIs), especially in cancer therapy. This work focuses on providing a complete and concise review of the synthetic tools available to thiolate cationic and anionic polymers, in particular chitosan (CTS) and hyaluronic acid (HA), respectively, drawing attention to the most successful procedures. Their chemical reactivity and most relevant properties regarding their use in anticancer formulations are also discussed. In addition, a variety of NP formation procedures are outlined, as well as their use in cancer therapy, particularly for taxanes and siRNA. It is expected that the current work could clarify the main synthetic strategies available, with their scope and drawbacks, as well as provide some insight into thiomer chemistry. Therefore, this review can inspire new research strategies in the development of efficient formulations for the treatment of cancer.

## 1. Introduction

**Figure 1:**
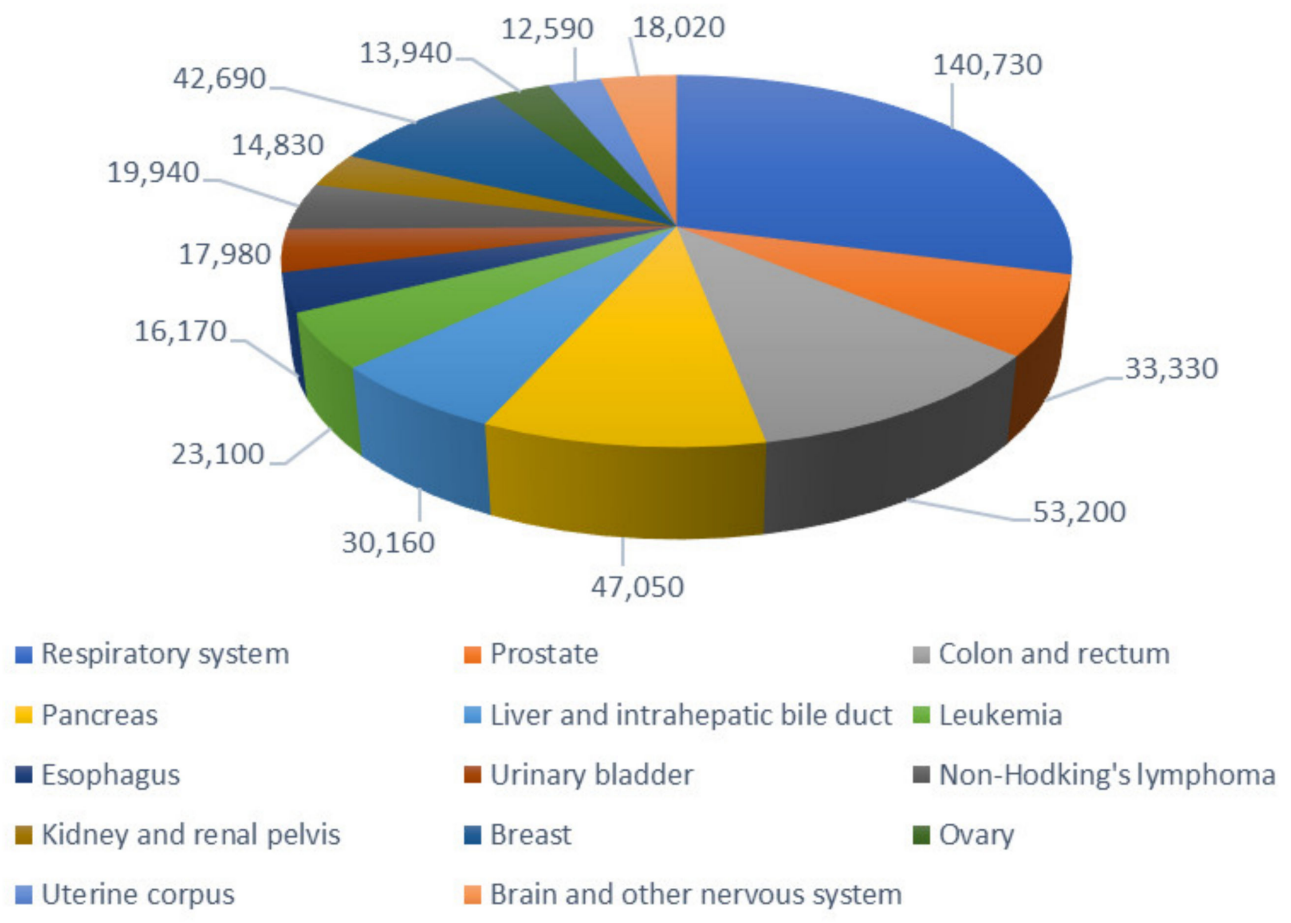
Estimated number of deaths by the main types of cancer in the United States, for both sexes, in 2020. Data obtained from [1].

Cancer is a major public health problem worldwide and is the second-leading cause of death in developed countries—only exceeded by cardiovascular diseases—which accounts for more than 8.8 million deaths per year. The lifetime probability of developing an invasive cancer is currently above 40% for men and close to that for women; in 2020, 1,806,590 new cancer cases and 606,520 cancer deaths were projected to occur in the United States (Figure 1) [1]. Cancer therapy is dictated by the cancer type, stage at diagnosis, and the patient’s tolerance to the prescribed therapy [2]. Chemotherapy serves as a method to block the expansion of growing neoplasm. Its results are enhanced when combined with other classical anticancer approaches [3]. However, the active pharmaceutical ingredients (APIs) for the treatment of cancer do not usually differentiate between healthy cells and cancer cells, as both of them are exposed to the cytotoxic effects of chemotherapeutic drugs [4]. In addition, cancer treatments generally involve the administration of relatively high doses of the drug in the hope that a portion, although minor, will go to damaged tissues [5]. Therefore, it is necessary to explore other therapeutic options that allow the drug of interest to accumulate in the cancerous tissue while decreasing the adverse reactions associated with its presence in healthy niches.

Nanotechnology has been extensively investigated for potential applications in the diagnosis and treatment of cancer, since it offers a suitable means of site-specific and/or time-controlled delivery of small- or large-molecular-weight drugs and other bioactive agents [1,2,3,4,5,6]. Drug-delivery systems (DDSs) based on polymer NPs have the potential to improve current disease therapies because of their ability to go through multiple biological barriers, overcoming the drawbacks of insoluble drugs, whereas their renal clearance will be reduced [4]. Additional benefits such as an increase in half-life, payload, and suppression of the side effects of toxic drugs are of great interest. Therefore, nanometric-sized systems are being studied for their use in either passive or active targeted cancer therapy. In cancer tissues, there are two relevant parameters that can act as internal stimuli in smart DDSs: the acidic pH [7,8] and the reductive environment found in tumors [9]. Both have been vastly explored as internal trigger for the release of payloads.

Thiolated polymers (thiomers) are biocompatible polymers that bear free or activated thiol groups covalently attached to them. They began to be explored in 1999 [10], and have since been used in multiple biomedical and pharmaceutical applications due to the close interest in their study among the research community [11,12,13]. These applications are connected with the presence of their thiol groups and their capability of conducting thiol–disulfide exchange reactions and oxidizing to disulfide bridges: from soft-tissue engineering [14] as biomaterial support for cartilage repair [15] and 3D bioscaffolds for cell culture [16,17] to antibacterial activity [18,19,20,21]. They are also prominent candidates for the formulation of controlled delivery carriers of APIs, whether for topical use [22], as dry powder inhalers [23], or in oral administration [24], among others. Their formulations can enclose small molecules such as methotrexate [25], sodium naproxen [26], isoniazid [23] docetaxel [27], and paclitaxel [28], as well as hydrophilic macromolecular drugs [29], but also peptides, RNA, and biomacromolecules like insulin [24], basic fibroblast growth factors [30], and siRNA [31].

In tackling the preparation of new thiolated polymers, derivatization of (semi)natural polysaccharides is an interesting approach because of their inherent biocompatibility, degradability, absence of toxicity, and chemical versatility. Due to their unique properties, chitosan (CTS) and hyaluronic acid (HA) stand out among the most promising polysaccharides studied for biomedical applications [11,12,13,32], and more recently, their thiolated derivatives are gaining attention. In this review, we will focus on the thiolation strategies of the major biocompatible polysaccharides CTS and HA as promising platforms for the development of nanoparticulated DDS in anticancer therapy. In particular, we will discuss the main approaches published regarding the delivery of selected anticancer APIs such as taxanes and siRNAs.

## 2. Cancer and Nanotechnology

**Figure 2:**
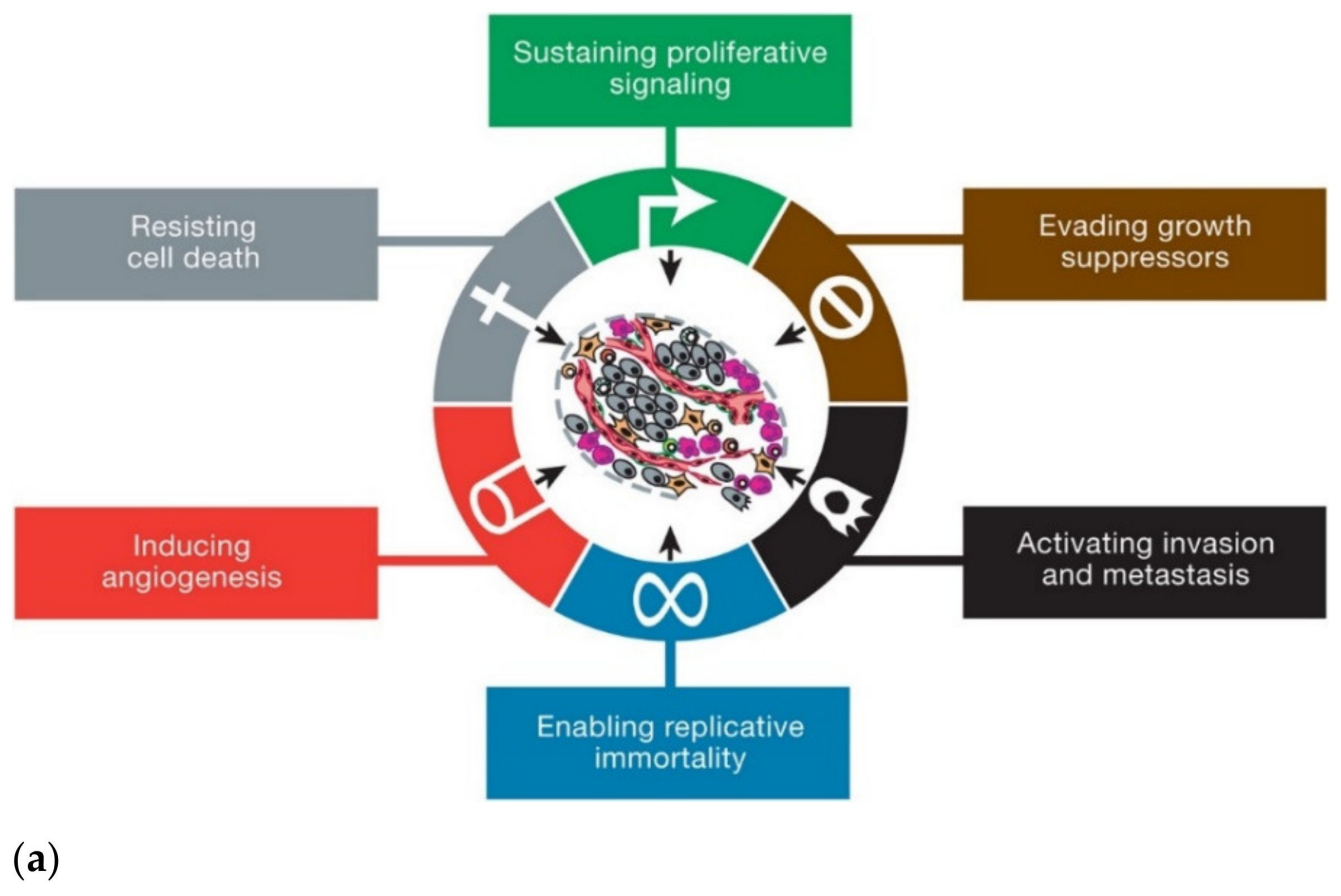

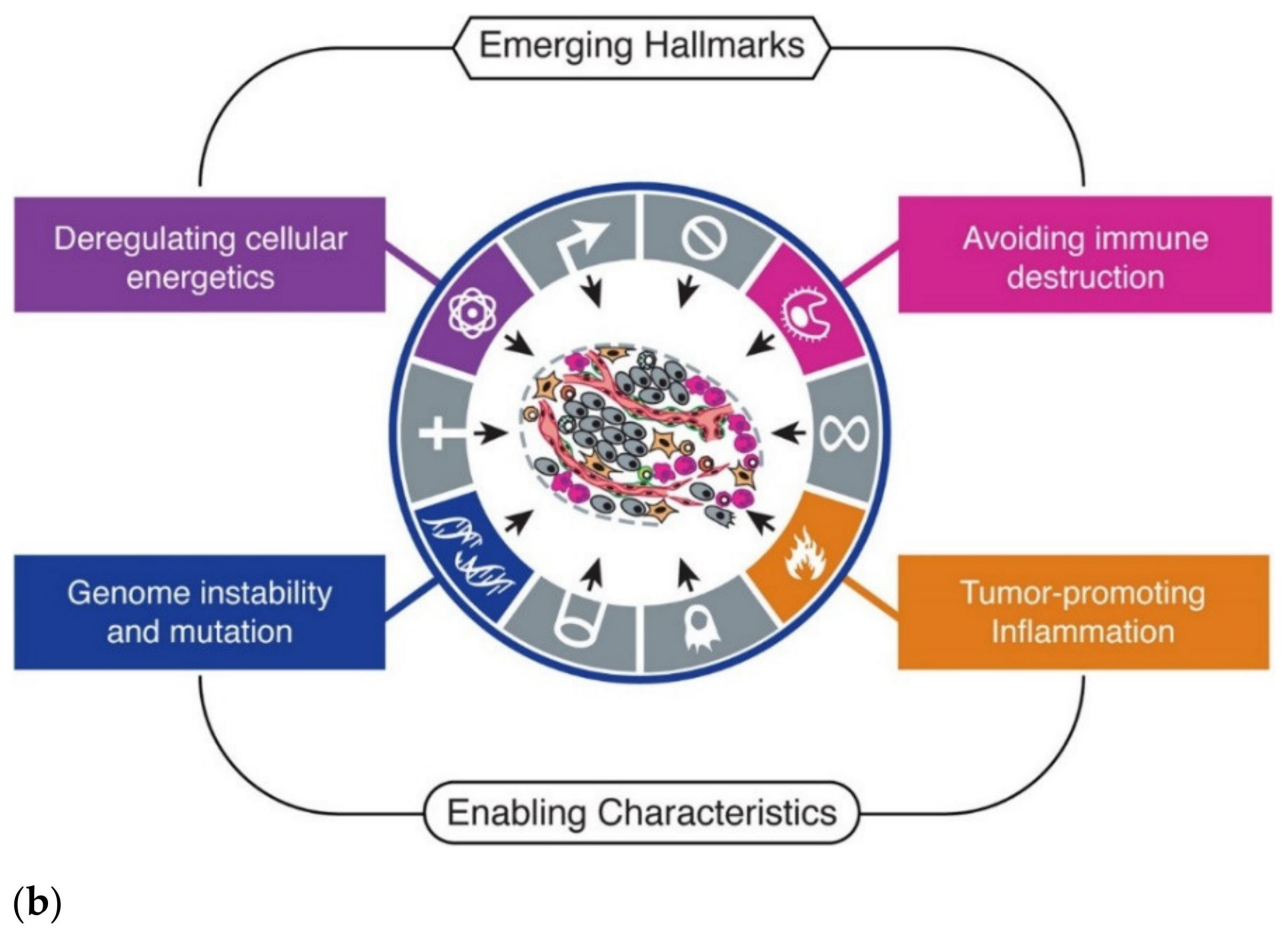
(**a**) The six hallmarks of cancer; (**b**) emerging hallmarks and enabling characteristics. Reprinted with permission from [33] (Copyright 2011 Elsevier Editorial).

Cancer has been one of the most studied pathologies in modern history. Molecularly, it is interesting to mention that, as of the year 2000, it was proposed that neoplastic diseases shared six characteristics that constitute their basic organizing principles, thus providing a logical framework to understand them even when the diversity among this group of pathologies is enormous. These peculiarities were baptized as “The Six Hallmarks of Cancer” (Figure 2a), and the consensus reached at that moment was that as healthy cells evolve gradually to a cancerous state, they acquire a succession of these hallmark abilities that allow them to behave as a prototypical tumor. The acquisition of such malignant properties during multistep tumorigenesis has been concluded to be favored by two enabling characteristics; that is, phenomena that create the necessary breeding ground for cancer cells to start developing: genome instability and mutation, and tumor-promoting inflammation [33].

Therefore, the six hallmarks of cancer can be defined as distinctive and complementary capabilities that enable tumor growth and metastatic dissemination [33]. They include: (1) sustaining proliferative signaling, (2) evading growth suppressors, (3) activating invasion and metastasis, (4) enabling replicative immortality, (5) inducing angiogenesis, and (6) resisting cell death. Nonetheless, an increased body of research during the past decades has suggested that there are not six hallmarks of cancer, but in fact eight, the list being completed with (7) deregulating cellular energetics and (8) avoiding immune destruction (Figure 2b) [33].

As far as cancer therapy is concerned, treatment is dictated by the cancer type, stage at diagnosis, and the patient’s tolerance to the prescribed therapy [2]. While surgery and radiotherapy are the primary treatment used for local and nonmetastatic cancers, other treatments can be employed such as chemotherapy, and hormone and biological therapies. Thus, for example, in the case of solid tumors, surgery is the local treatment of choice, as the damage is confined to a limited area of the body. However, most patients require the combination of two or more therapeutic treatments due to the potential spread of the disease, as well as to effectively prevent the evolution of the disease from early to advanced stages. Chemotherapy works by inhibiting the division of rapidly growing cells, and combined with surgery or radiotherapy, the effectiveness of these treatment modalities are increased [3].

Although chemotherapy is the main treatment for cancer patients, the active pharmaceutical ingredients (APIs) used do not differentiate between healthy cells and cancer cells. Both of them are exposed to the cytotoxic effects of chemotherapeutic drugs, and consequently, the drugs interfere the growing pattern of normal cells with fast proliferation rates, such as the hair follicles, and bone marrow and gastrointestinal tract cells, and provoke long-term toxic effects on the heart, lungs, and kidneys [4]. Typical harmful side effects associated with chemotherapy, such as nausea, vomiting, immune suppression, hepatotoxicity, nephrotoxicity, memory loss, anemia, and even death, are rooted in the use of such nonspecific therapeutic systems.

Therefore, and regarding drug administration, the methods used traditionally have been limited to making the drug accessible to the bloodstream, relying on the irrigation and the drug affinity for the tissues for the access to the target. In fact, bioavailability is still measured from drug levels in the bloodstream, not in the target surroundings. In many cases, only a small portion of the administered drug reaches the tumor site [6]. As a consequence, cancer treatments generally involve the administration of relatively high doses of the drug in the hope that a portion, although minor, will go to damaged tissues [5]. Thus, there is a need to increase the drug concentration in the cancerous tissue while reducing the side effects associated with chemotherapeutic molecules. This requirement is even more compelling in the case of highly toxic anticancer drugs, which may also present physicochemical and stability features that are too deficient. In addition, this becomes complicated when considering that even when the drug reaches the tumor, cancer cells can develop drug resistance. For instance, P-glycoprotein (P-gp, where “P” refers to permeability) has been documented to be overexpressed in various drug-resistant tumors, thereby enabling direct drug efflux (meaning it works as a drug efflux pump) and limiting intracellular accumulation of several anticancer agents [34]. Thus, the indiscriminate destruction of normal cells, the toxicity of conventional chemotherapeutic drugs, as well as the development of multidrug resistance, support the need of finding new effective targeted treatments based on the differences found in the molecular biology of the tumor cells.

Significant efforts have been devoted to take advantage of the potentials of nanotechnology in drug delivery, since it offers a suitable means of site-specific and/or time-controlled delivery of small- or large-molecular-weight drugs and other bioactive agents. Nanotechnology has also been extensively studied for potential applications in the diagnosis and treatment of cancer [1,2,3,4,5,6].

Pharmaceutical nanotechnology focuses on formulating APIs into biocompatible nanoforms in which the drug is dissolved, entrapped, encapsulated, or attached to a nanoparticle matrix. The nanoscale size of these delivery systems is the basis for many of these advantages. Several types of nanoparticulate systems have been attempted as potential DDSs, including biodegradable polymeric nanoparticles (NPs), polymeric micelles, solid nanoparticles, lipid-based nanoparticles (e.g., solid lipid nanoparticles (SLNs), nanostructured lipid carriers (NLCs), and lipid drug conjugates (LDCs)), nanoliposomes, inorganic nanoparticles, dendrimers, magnetic nanoparticles, ferro-fluids, and quantum dots [35]. Among the most widely investigated nanocarriers in cancer detection and cancer therapy are carbon nanotubes, micelles, dendrimers, polymeric nanoparticles, liposomes, nanoshells, and polymer–drug conjugates/proteins.

Polymer NPs have gained significant attention among the numerous nanotechnology approaches, as is stressed by the fact that over 90% of the scientific articles published on cancer therapeutics in the last decade were based on the use of such systems [4]. DDSs based on polymer NPs have the potential to improve current disease therapies because of their ability to overcome multiple biological barriers and release a therapeutic molecule within the optimal dosage range. They are also capable of overcoming the drawbacks of insoluble drugs. This is the case for anticancer molecules such as camptothecin [7], doxorubicin [36], and the taxanes paclitaxel [37] and docetaxel [27]. Additional benefits such as an increase in half-life, payload, and solubility of APIs can be attained. Being embedded into the nanoplatform conjugate, chemotherapeutic drugs can be transported to tumors without damaging healthy tissues and, therefore, a drop in their toxicity profile in the human body is expected to occur.

**Figure 3:**
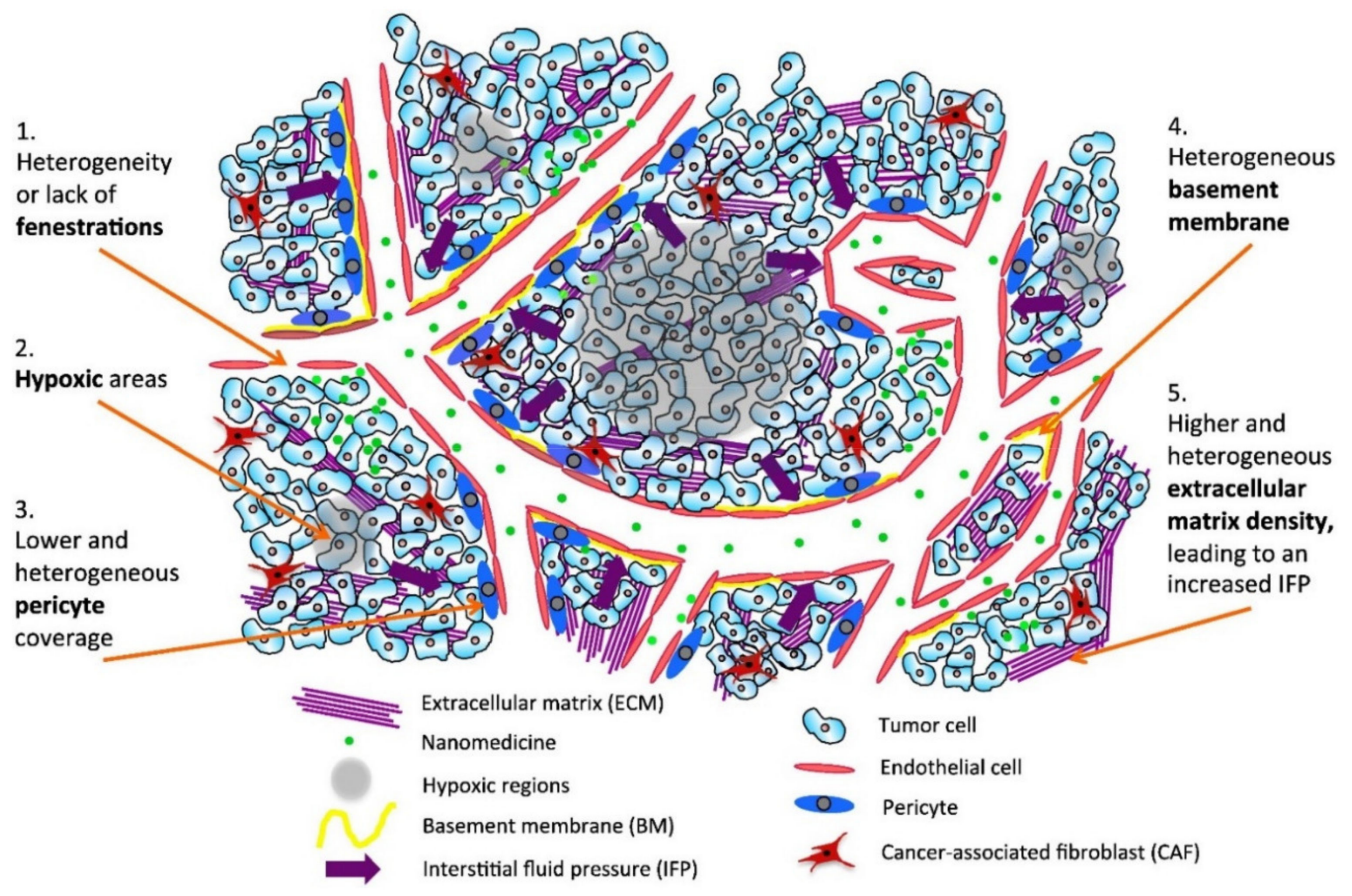
Schematic representation of the passive targeting of nanomedicines (EPR effect) in human tumors. Numbers represent some major differences found in tumor microenvironments of human cancers in contrast with murine tumors. Reprinted with permission from [39] (Copyright 2016 Elsevier Editorial).

Consequently, nanometric-sized systems have become the option of choice, and they are being investigated for their use in either passive (by enhanced permeability and retention) or active (by the functionalization of the surface of the carriers) targeted cancer therapies. Angiogenesis during tumor growth results in a defective hypervascularization and a deficient lymphatic drainage system, which has given rise to the concept of passive targeting of NPs to tumors through the “enhanced permeability and retention” (EPR) effect [4,38]. Passive tissue targeting uses the increased permeability of tumor vasculature and the poor lymphatic drainage of tumors (EPR effect; Figure 3) [39], allowing drug-delivery nanocarriers (cutoff size of >400 nm) to accumulate and diffuse preferentially in the vicinity of tumors, with the desirable release of the chemotherapeutic agents in the tumor.

However, not only the encapsulation, but also the release of drugs at the target tissue is vital in drug delivery. By introducing responsive groups, the carrier could release the drug under specific stimuli. In order to ensure controllable and optimal releasing at desirable sites, a variety of “stimulus-responsive” nanoparticulated DDSs have been designed [40,41]. Internal (or endogenous) stimulus arises from variation in a target site parameter, such as relative changes in pH; different expression of a specific enzyme, factors, or other molecules; and abnormal redox balance [42]. In cancer tissues, there are two relevant parameters that can act as internal stimuli: the acidic pH and the reductive environment found in tumors. Thus, compared with healthy tissues, lower pHs (6.2–6.9) of the extracellular matrix have been found in cancerous cells because of the Warburg effect [43]. The differences in pH have been vastly explored as an internal trigger for the release of payloads [7,8]. In addition, pH differences between intracellular endosomes/lysosomes (with pH of 4.0–6.0) of normal and cancer cells is extraordinary [44]. Redox potential is another internal stimulus for responsive release of drugs [9].

Glutathione (GSH), a tripeptide constituted by L-γ-glutamyl-L-cysteinyl-glycine, is involved in the formation and lysis of disulfide bridges; it serves as a general reductant for cells, and its intracellular concentration is 1–10 mM in mammalian cells [45]. Glutathione exists in reduced (GSH) and oxidized (glutathione disulfide, GSSG) states, where the GSH/GSSG system is the major redox couple in animal cells [14]. GSH plays several vital roles in maintaining the bioactivity of cells, including antioxidation, maintenance of the redox state, modulation of the immune response, and detoxification of xenobiotics [42]. The ratio of the GSSG/GSH couple can serve as an important indicator of the cellular redox environment [46]. Intracellular concentration of GSH is hundreds to thousands of times higher than that in ECM [47], and more importantly, elevated levels of GSH are found in tumor cells; for example, in bone marrow, breast, colon, larynx, and lung cancers, to minimize radical damage from oxidative stress [46]. Thus, the disulfide crosslinking reaction has an advantage in the development of cancer therapies over other chemistries, since redox potential is an internal stimulus for responsive releasing of delivery system.

With all of this in mind, it is no surprise that thiomers (that is, polymers that have undergone modifications regarding the addition of thiol groups) have broken though as avant-garde approaches in this field. Several studies agreed that their multiple properties make them perfect candidates to take part in nanoformulations that act as drug-delivery systems for compounds with major difficulties in administration, especially in anticancer therapy.

## 3. Thiomers: Reactivity and Distinguished Properties

Thiolated polymers (thiomers) are macromolecules with free and exposed thiol groups on the surface of the polymeric backbone, covalently attached by different synthetic routes. Thiomers came into the research arena in the 1990s [10], and have since found multiple applications. For example, thiomers display a great cation binding capacity [48,49], demonstrated for metal ions such as Au(III), Pd(II), Pt(IV) [50], As(III), As(V) [51], Hg(II), Cu(II), and Ni(II) [52], as well as an efficient Pd carrier [53]. They are also of utility in the fabrication of colorimetric sensors for Hg^2+^ detection at trace levels [54]. As mentioned before, thiomers are of special interest in biomedical applications; for example, as 3D bioscaffolds [14,15,16,17]. Some of them exhibit antibacterial activity. Thus, *N*-acyl thiolated CTS has demonstrated to be a rewarding target group for highly efficient, biocompatible and cost-effective antimicrobial compounds [18]. Therefore, the thiolated low-molecular-weight CTS–thioglycolic acid conjugate (CTS–TGA) was proposed as a promising and effective broadband antimicrobial compound [21]. In addition, 3D sponges composed of chitosan thiolated with 11-mercaptoundecanoic acid (CTS–MUA) showed high antibacterial activity and specificity against *Pseudomonas aeruginosa,* equivalent to conventional antibiotic drugs [19]. Such sponges were also relevant as a potential environmental pollution remediation, since they exhibited high adsorption capacity for methyl orange, the chosen model organic pollutant [19]. The antibacterial effect of CTS–MUA against *Echerichia coli* was confirmed and showed better antibacterial ability than pristine chitosan [20].

Specifically, and due to their exceptional properties, thiomers have shown to be potentially useful materials for the synthesis of nanostructures specialized in the delivery of multiple compounds via different routes of administration. As mentioned above, enclosed molecules vary from peptides or siRNA to some biomacromolecules or hydrophilic drugs. For example, the use of CTS thiomers for the formation of peptide nanocarriers was demonstrated by Cheng et al. [55]. The prepared thiolated glycol–CTS succinate formed ionic complexes with the positively charged melittin, an amphipathic peptide used in cancer therapy. The dual secured nano-sting (DSNS) was prepared through the combination of zwitterionic interactions and disulfide bonds. The lethal action of melittin-loaded DSNS for MCF-7, HCT-116, SKOV-3, and NCI/ADR-RES (multidrug resistant) cancer cells was confirmed, as the system could kill almost 100% of them without showing any hemolytic effect.

Before going into the methods for preparing thiomers, their reactivity and properties are briefly discussed below.

**Figure 4:**
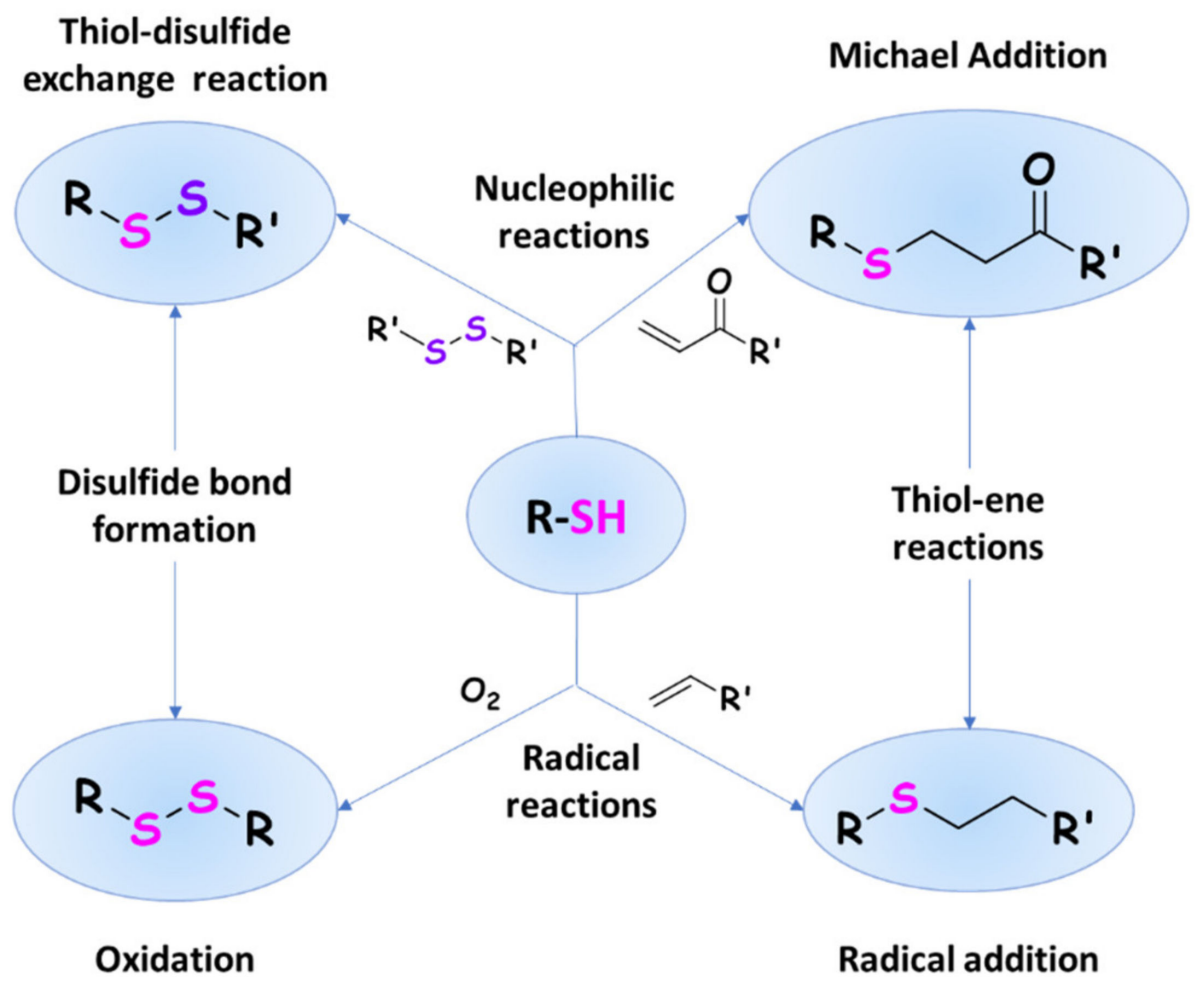
Selected examples of reactivity of thiomers.

In general terms, thiol groups can react by means of two types of mechanisms: either nucleophilic or radical mediated reactions, some of which are recorded in Figure 4. As nucleophilic examples, the thiol–disulfide exchange reaction and Michael reaction are especially relevant, and the oxidation and radical-initiated thiol–ene click reaction belongs to those that exhibit a radical mechanism. Thiolate anions (R-S^−^) are the reactive species involved in nucleophilic reactions, and hence, the p*K*_a_ of the thiol moiety is a vital parameter for these chemical modifications [56,57]. On the other hand, thiyl radicals (R-S^•^) are the reactive species in the oxidation of thiol moieties by oxygen, as well as in thermal or photo-initiated thiol–ene reactions [58].

### 3.1. Thiol Oxidation and Disulfide Bond Formation: In Situ Gelling Properties

One of the reactions in which thiomers intervene, either on purpose or as a side reaction, is the formation of disulfide bonds. Disulfide linkages play a key role in numerous properties found for thiolated polymers such as mucoadhesiveness, in situ gelling features, permeation enhancing, and efflux-pump inhibition [59], and are of particular interest for bioconjugation [60], controlled drug delivery [26,30,61], and cell encapsulation [16,17,62], due to the ability of cells to cleave disulfide bonds by secreting natural reductants such as glutathione.

The preparation of materials with disulfide bonds in their structure can take place by a radical or nucleophilic mechanism (Figure 4), the first being preferred. Thiol oxidation is a multistep process that requires the initial formation of thiolate anions (R-S^−^) that react with molecular oxygen to generate the reactive radical species (R-S^•^) [63]. Thus, the formation of disulfide bonds by both mechanisms is critically dependent on the acidic character of the sulfhydryl moiety. Understanding the balance between thiol and thiolate anion residues at different pHs allows a deeper understanding of such properties.

**Figure 5:**
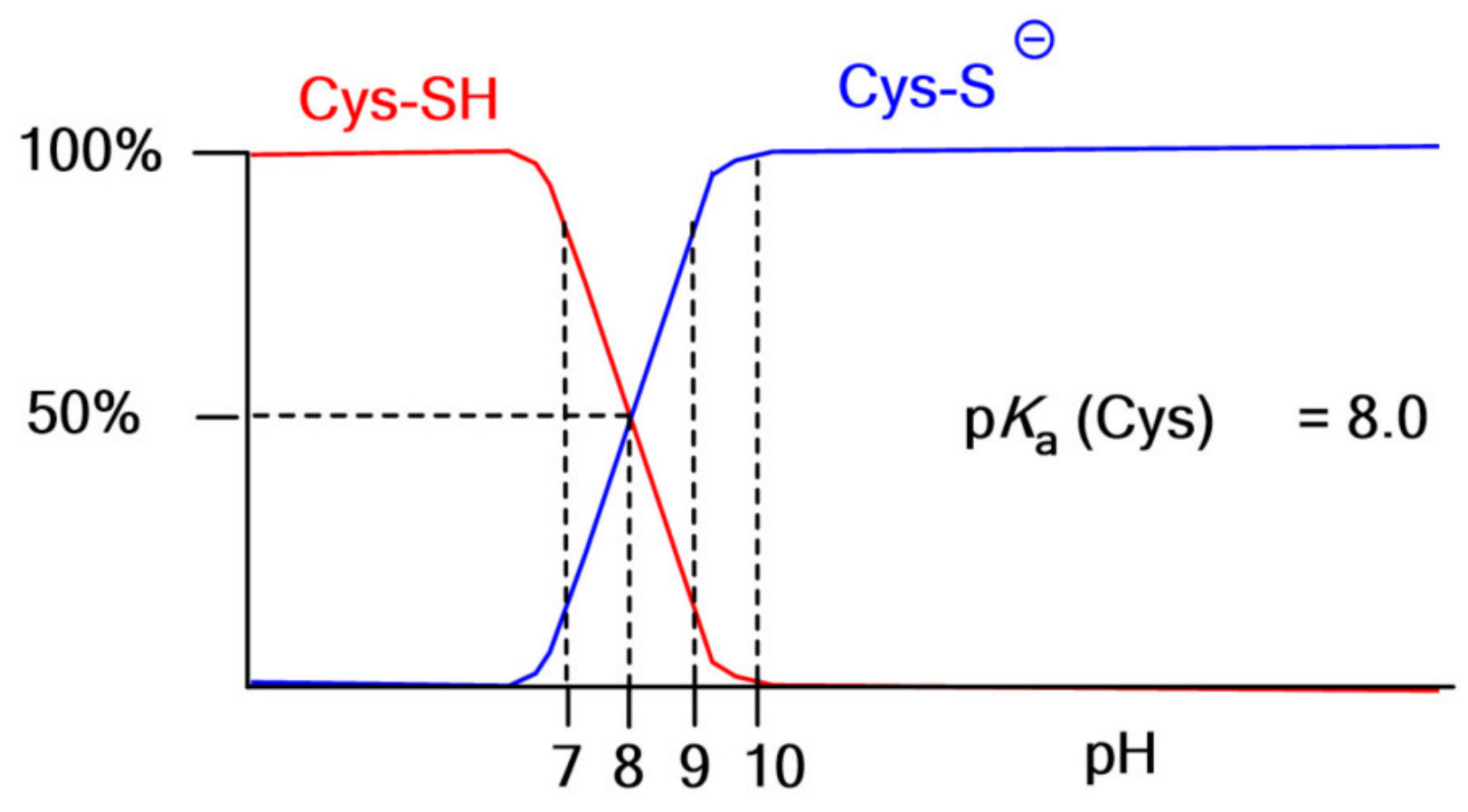
Representation of the concentrations of RSH and RS^−^ (in percentages, calculated from the Henderson–Hasselbalch equation) for Cys based on its p*K*_a_.

The ratio of thiols to thiolate anions is governed by the p*K*_a_ of the sulfhydryl group so that the lower the p*K*_a_, the higher the percentage of R-S^−^ at a fixed pH and, therefore, its reactivity. Taking L-cysteine (p*K*_a_ = 8.0) as an example and according to the Henderson–Hasselbalch equation (Equation (1)), once pH values pass from p*K*_a_-1 (from pH 7 and above), a significant [R-S^−^] is found in the medium, and it increases steadily until the values are close to 100%, when the pH is two units above the value of p*K*_a_ (pH = p*K*_a_ + 2 = 8 + 2 = 10) ratio [(R-S^−^/R-SH) = 100/1 at pH 10] (Figure 5).

(1) $$\mathrm{pH}=pK_{a}+\log\frac{\left[R-S^{-}\right]}{\left[R-\mathrm{SH}\right]}$$

Since thiols have a p*K*_a_ of ∼8–10, this oxidation process is, in general, a very slow reaction under physiological conditions (pH 7.4). However, the p*K*_a_ of a thiolated residue is influenced by neighboring groups, and hence by their incorporation into a polymer backbone. For example, Bermejo-Velasco et al. [63] have investigated the in situ gelling properties of HA thiomers, a common feature of thiolated polymers that is linked to disulfide formation and which can lead to a boost in the mechanical stability of the material. In general, disulfide bond formation by a radical mechanism does usually require long periods and is addressed either at basic pH [64], with the consequent increase on [R-S^−^] in the media, or using oxidants such as H_2_O_2_ or iodine [65,66]. Achieving it at physiological pH and at a convenient tempo is of great interest for biological applications. Specifically, they have studied the effect that the presence of substituents in the β-position to the thiol group in the derivatization residue has in disulfide formation. They demonstrated that electron-withdrawing groups at the β-position reduced the p*K*_a_ of the thiol group to 7.0 for HA-Cys, 7.4 for *N*-acetylcysteine (HA-NAC), and 8.1 for HA-3-mercaptopropanoic acid (HA-MPA) derivatives, respectively. Thus, the thiolate anion (R-S^−^), the concentration of which controls the kinetics of disulfide formation, would be the predominant species in the acid–base equilibrium for HA-Cys, and accounts for 50% for HA-NAC at pH 7.4, in agreement with the Henderson–Hasselbalch equation. It was not surprising that the authors then found that HA-Cys and HA-NAC formed hydrogels at pH 7.4, with the procedure being remarkably quicker than the former (3.5 min for HA-Cys compared to 10 h for HA-NAC). The other thiolated polymer, HA-MPA, did not form any gel at pH 7.4 even after 24 h.

When thiol oxidation is a reaction to prevent, it should be taken into account that thiolated polymers are susceptible to oxidation in aqueous media at pH > (p*K*_a_-2), being more pronounced at higher pHs [13,63]. This side reaction can be induced by oxygen [16,25,67], being available in most body fluids and aqueous media, or by oxidizing agents [68]. The addition of antioxidants or reducing agents at the end of the thiolation reaction such as 2-mercaptoethan-1-ol [69], dithiothreitol (DTT) [20,30], or tris(2-carboxyethyl)phosphine (TCEP) [18,70] can cleave/reduce disulfide bond formation.

Another alternative to prepare disulfide linkages is by the thiol–disulfide exchange reaction (Figure 4). As mentioned above, this is a nucleophilic procedure and is conducted by the nucleophilic attack of the thiolate anion (R-S^−^) over an already-formed disulfide linkage (R’-S-S-R’).

**Figure 6:**
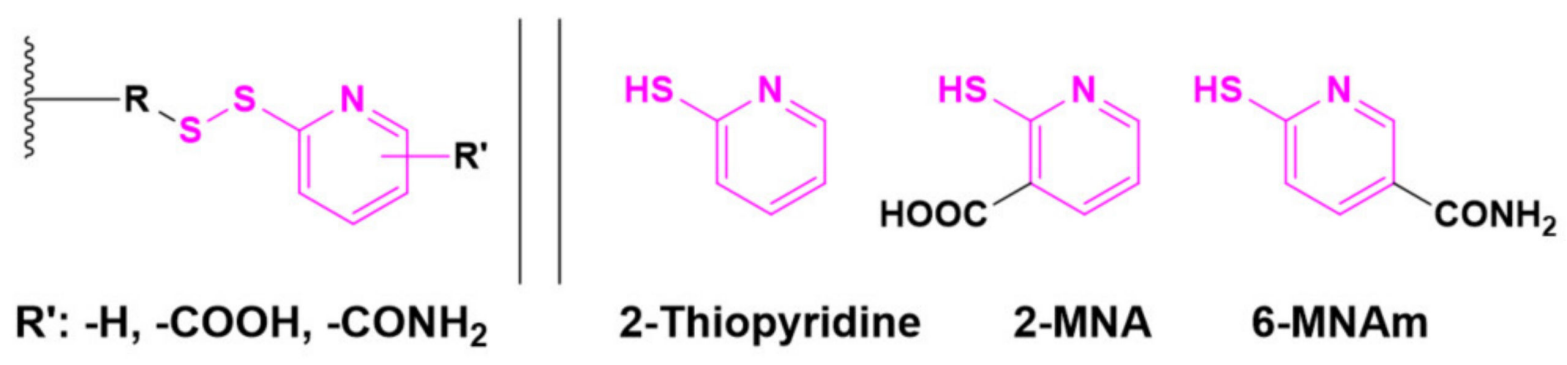
General structure of thiomers with protected-preactivated sulfhydryl groups with 2-pyridylthio substructure (2-MNA: 2-mercaptonicotinic acid; 6-MNAm: 6-mercaptonicotinamide).

Free thiols from thiomers can be protected while preactivated with a 2-pyridylthio substructure, such as those derived from 2-mercaptopyridine, 2-mercaptonicotinic acid, (2-MNA) and 6-mercaptonicotinamide (6-MNAm), with the latter two being preferred to the former due to safety reasons (Figure 6) [63].

This *S*-protection not only provides stability for their extemporaneous oxidation, but also increases their reactivity compared to the unprotected counterparts when they meet the cysteine-rich subdomains in the human body, thus improving their stability and reactivity [71]. Consequently, they are known as preactivated thiomers. This augmented reactivity responds to the electron-withdrawing effect of the π-deficient ring, which promotes the lysis of asymmetric disulfide linkages, so that the formation of more stable disulfides, with electron-donating groups, is favorable. Hence, the thioamide 2-mercaptopyridine, 2-MNA, or 6-MNAm can be easily cleaved off [72].

### 3.2. Thiol–Ene Reactions

The relatively weak sulfur–hydrogen bonds of thiols, as well as the nucleophilic nature of thiolate ions (R-S^−^), are responsible for the participation of thiols in a plethora of chemical reactions that occur under mild conditions with almost quantitative yields. Two relevant thiol reactions, which are englobed under the term thiol–ene reactions, describe the modifications of thiols with a wide variety of unsaturated functional groups: on one hand, the radical addition of thiol to electron-rich/electron-poor carbon–carbon double bonds; and on the other, the catalyzed anionic thiol Michael addition to electron-deficient carbon–carbon double bonds (Figure 4).

Reactions of thiols with enes, whether proceeding through a radical or anionic mechanism, have many of the attributes of click reactions [73], and accordingly, are now routinely referred to as thiol click reactions in the literature. Among their most outstanding properties, it is worth mentioning that: (1) they are facile synthetic strategies in which a range of easily obtainable starting materials participate, many of them being commercially available (both thiols and enes); (2) they are highly efficient reactions (quantitative conversions), easy to perform under mild conditions, that do not originate byproducts and display fast reaction rates; (3) they are tolerant to many different reaction conditions/solvents, for example, being insensitive to water, and more significantly, to ambient oxygen; (4) they can be conducted either in bulk or in environmentally benign solvents over a large concentration range, and only small concentrations of relatively benign catalysts are needed; (5) a clean-up step is essentially unnecessary; (6) these synthetic processes follow clearly defined reaction pathways that yield a single regioselective product; and (7) the orthogonality of the reactions and the compatibility of the processes with numerous functional groups and biological processes make them especially attractive for biomedical applications.

**Figure 7:**
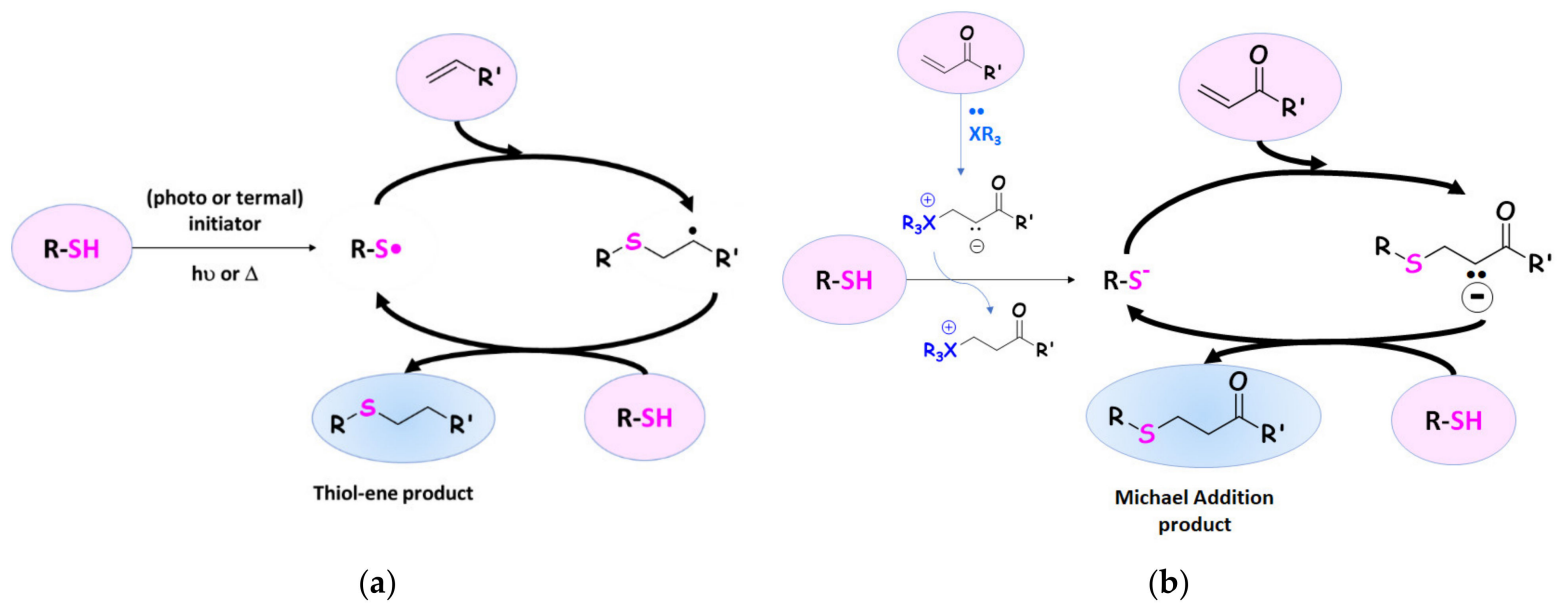
Mechanisms of (**a**) radical- and (**b**) nucleophile-initiated thiol–ene additions.

Any nonsterically hindered terminal ene can participate in the radical-mediated thiol–ene process, with electron-rich (vinyl ether) and/or strained enes (norbornene) reacting more rapidly than electron-deficient enes. The relative reactivities (in order of decreasing reactivity) of enes toward radical thiol–ene reactions are as follows: norbornenes > vinyl ethers > methacrylate > acrylonitrile > styrene > maleimides > conjugated dienes [74]. Thiol–ene reactions can be induced by thermal or photochemical initiators (Figure 7a), and it was found that those using photochemical conditions lead to faster and, in many cases, quantitative conversions [75]. The ideal thiol–ene radical reaction revolves around the alternation between the thiyl radical and the carbon-centered radical derived from the ene; conversions near 100% are achieved, and the net reaction is simply the combination of the thiol and the ene moieties to render the alkylthioether derivative. When photopolymerizations based on thiol–ene reactions are conducted, highly uniform polymer networks are achieved, promoting unique capabilities related to spatial and temporal control of the click reaction [76]. Photo-initiated thiol–ene reactions are frequently used for the synthesis of hydrogel networks in the biomedical field [77].

Another interesting nucleophilic reaction in which thiol groups participate is the Michael addition, commonly used to crosslink thiomers to improve the mechanical properties of, for example, 3D scaffolds and hydrogels [11,78,79]. It is also of interest in the anchoring of APIs to DDSs such as the monoclonal antibody Cetuximab [80].

Mercapto groups act as Michael donors and react with activated olefines, namely α,β-unsaturated carbonyl groups such as maleimides, vinyl sulfones, acrylates, and methacrylates (cited from highest to lowest reactivity). The reaction is catalyzed by a nucleophile (Figure 7) that generates the thiolate anion, which is the nucleophilic species. R-S^−^ will react with the β carbon from the α,β-unsaturated carbonyl moiety [11,15,78,79]. The catalysts are nucleophilic species such as amines [81] or alkylphosphines [82], which have been demonstrated to be extremely efficient in speeding up thiol–Michael addition reactions.

### 3.3. Properties for Biomedical Applications

However, high reactivity of thiol groups does not always correlate with enhanced physiological properties, such is the case of their mucoadhesion behavior to biological surfaces like mucins or keratins. It should be considered that free unprotected thiol groups react with disulfide bonds of proteins by the thiol–disulfide exchange reaction, whereas *S*-protected thiol groups react with free thiol groups of proteins.

When bringing the thiolated polymers into contact with the mucosa, their pronounced mucoadhesiveness can be explained by disulfide bond formation between the sulfhydryl groups of the polymer and cysteine-rich subdomains of glycoproteins in the mucus layer [83]. Thus, a correlation between the amount of conjugated thiol groups in the polymer and the mucoadhesive bond strength has been revealed [84]. Furthermore, and as demonstrated for thiomers from poly(acrylic acid) (PAA), the extent of immobilization of thiol moieties is augmented when thiolation is conducted in organic solvents, as is their mucoadhesive character [85]. Nonetheless, thiol content is not the only parameter involved in mucoadhesive capacity. Rapid formation of disulfide bridges with thiol groups on the surface of the mucosal tissue is not advisable because the thiomer-based drug carrier will be eliminated faster by the mucus turnover process [13]. Additionally, adhesion improves with flexible polymer chains so the entanglement between the underlying mucus substructures within the polymer matrix is facilitated [10]. In this case, both the entanglement and the mucosal network structure will take place with the formation of disulfide bonds by means of the reaction of the thiomers with the cysteine subdomains within the mucosal layer. This reinforcement is prevented when thiomers with poorly reactive mercapto groups are used. Therefore, thiol moieties must have the appropriate reactivities, the most favorable p*K*_a_ values being those near 8, as it is also preferred by nature in the form of Cys. Lastly and not surprisingly, a sufficient hydration of the thiolated polymer is a prerequisite for the mobilization of polymer chains. Thus, the use of hydrophilic thiomers that bear functional groups capable of forming hydrogen bonds greatly improved this property [86]. It is worth mentioning that preactivated thiomers have demonstrated an ability to exert improved mucoadhesion in several polymers and applications [87,88,89].

Another of the exceptional characteristics attributed to thiomers is their behavior as a permeation enhancer through the opening of tight junctions (TJs) of epithelial cells caused by the interaction of thiomers with cysteine-bearing membrane receptors and enzymes [90]. When used in oral DDSs, they can adhere to the mucosa of the intestine [91], thereby inhibiting the enzymatic degradation of APIs and enhancing drug permeation via opening TJs of epithelial cells. Zhang et al. [92] proposed that, by interacting with a highly expressed cysteine-rich membrane receptor like epidermal growth factor receptor (EGFR), thiomers activate Src phosphorylation and further disrupt claudin-4, which results in the TJs opening eventually. The effect was found to be reversible, and the integrity of the epithelium was restored when the contact with the polymer was discontinued [93]. In permeation studies of model compounds, thiomers display comparatively more prolonged effect and lower toxicity than low-molecular-weight permeation enhancers [94]. The effect could be further improved by the addition of the permeation mediator GSH [94,95]. However, this property can drop substantially for thiomers with free sulfhydryl groups in a normal intestinal environment since, as mentioned above, thiol groups are prone to be oxidized under such conditions. Preactivated thiomers, with the protective 2-pyridylthio-based moiety, impede the premature oxidation of sulfhydryl groups so their functionality is preserved. They have demonstrated great merits in oral delivery of protein/peptide drugs by triggering the opening of tight junctions of epithelial cells [96].

A thiomer-based DDS can be the option of choice when dealing with the issue known as multidrug resistance (MDR) in anticancer therapy. MDR is a phenomenon whereby cells confer resistance to structurally and functionally unrelated APIs, such as chemotherapeutic drugs, antibiotics, cardiac glycosides, and antiretroviral APIs. Multidrug-resistant proteins like P-glycoprotein and MRP1 are responsible for the reduced bioavailability of such drugs in numerous cases of cancer. The role of in drug resistance was first reported by Juliano and Ling [97]. Elevated P-gp expression has been documented in various drug-resistant tumors, thereby enabling direct drug efflux, and limiting intracellular accumulation of anticancer agents that serve as substrates for this transporter [34]. Thiomers can form disulfide bonds with the cysteine subunits present in P-gp and MRP1 transmembrane-channel-forming structures, and thereby inhibit the activity of these transporters (inhibition of efflux pumps) [12,98].

Additionally, API-loaded thiolated carriers experience enhanced absorptive endocytosis by disulfide formation with exofacial thiols of transmembrane proteins [31,99,100]. 

The use of thiolated materials as a constituent of NPs can make the nanocarrier reactive under the environments found in cancer tissues; hence, thiomer-based nanocarriers behave as smart DDSs, not only for conventional chemotherapeutics, but also for other novel APIs like small interfering RNA (siRNA), as will be discussed below. However, to keep the nanocarrier stable in the blood circulation before it reaches the target, stable and strong interactions are needed. Disulfide bonds are amply stable in the blood circulation, but they are prone to rapid cleavage under a reductive environment through the fast and readily reversible thiol–disulfide exchange reactions [13]. Thus, thiomer-based, drug-loaded NPs crosslinked by disulfide linkages could maintain their integrity in the bloodstream and were later disintegrated in tumor surroundings by means of GSH [36,101].

It is evident that thiomers have demonstrated promising properties that can make them a useful tool in the development of novel formulations for clinical use. Pathologies that are nowadays treated with rather toxic compounds (or those that do not even have an effective treatment) can find new horizons to tackle in these materials. Without a doubt, anticancer therapy is one of the main fields in which thiolated polymers are breaking through more evidently, which certainly will continue in the near future.

## 4. Thiolation of (Semi)Natural-Occurring Polysaccharides

Although thiol chemistry is already well developed, the emergence of these new materials has prompted research in this field, with the design of a wide variety of materials that have been prepared using a plethora of synthetic procedures, many of them centered on the derivatization of (semi)natural-occurring polymers such as chitosan and hyaluronic acid.

### 4.1. Strategies for the Thiolation of Multiamino Polymers

The most common way to prepare thiolated polymers is by derivatization of (semi)natural polymers such as chitosan, hyaluronic acid, cellulose, dextran, cyclodextrins, etc., the former being the polysaccharide on which more research has been conducted.

The accumulated information about the physicochemical and biological properties of chitosan (CTS) led to the recognition of this polymer as one of the most promising material for DDSs [35]. The polysaccharide CTS, a weak cationic polysaccharide composed of randomly distributed β-(1-4)-linked d-glucosamine and *N*-acetyl-d-glucosamine repeating units, is a copolymer prepared from renewable resources. It can be obtained from the partial deacetylation of the second most important natural polymer in the world: chitin or poly(*N*-acetyl-β-d-glucosamine). Its abundance in marine crustaceans, such as shrimp and crabs, makes it a commercial product with global impact in polymer science. CTS bears exceptional properties such as nontoxicity, biocompatibility in vitro and in vivo, degradability, and a broad activity spectrum against Gram-positive/negative bacteria and fungi [18]. On the other hand, CTS can interact with negatively charged polymers, macromolecules, and even with certain polyanions upon contact in an aqueous environment. These interactive forces and the resulting sol–gel transition stages have been exploited for nanoencapsulation purposes [102]. Moreover, CTS has the special feature of adhering to the mucosal surfaces within the body, a property leading to the attention to this polymer in mucosal drug delivery [103]. The potential of CTS for this specific application has been further enforced by the demonstrated capacity of chitosan to open tight junctions between epithelial cells though well-organized epithelia [104]. Consequently, due to the interesting biopharmaceutical characteristics of this polymer, accompanied by its well documented biocompatibility and low toxicity, numerous articles on the potential of chitosan for pharmaceutical applications have already been published [105,106,107,108].

**Figure 8:**
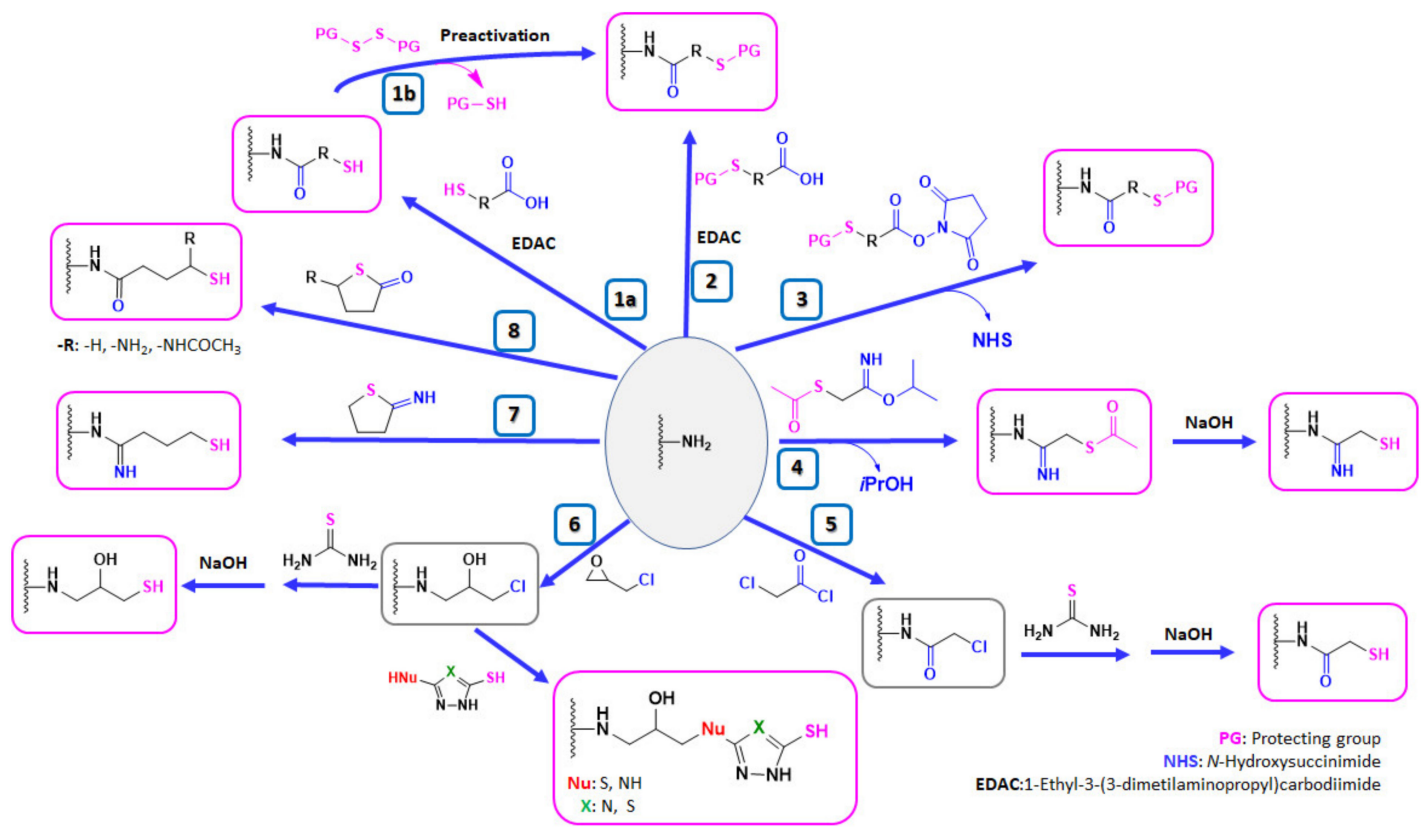
Reported routes to thiolated multiamino polymers.

From the synthetic point of view, the presence of the nucleophilic amino group in the CTS structure is capable of imparting distinctive reactivity to the material, which differentiates it from other polymers, making it a unique polysaccharide. Thus, the amino groups of chitosan are responsible for most of its derivatization reactions on the way to thiolated polymer formation; Figure 8 displays the most common synthetic techniques used. CTS, poly(lysine), and polyethyleneimine (PEI) are examples of cationic multiamino polymers, and although the chemistry described next has been developed using as starting material CTS or its derivatives, it could be extended to other multiamino polymers.

#### 4.1.1. Coupling Reaction of Multiamino Polymers and Mercaptocarboxylic Acids

Thiol-bearing compounds can be covalently linked to multiamino polymers via the formation of amide or amidine bonds, the former being the preferred strategy. There are several approaches developed with such targets that will be described next.

The most common reaction to include sulfhydryl groups into multiamino polymers is the coupling reaction between the amino groups and the carboxylic acid groups from mercaptocarboxylic acids mediated by a water-soluble carbodiimide: the 1-ethyl-3-(3-dimethylaminopropyl)carbodiimide (EDAC, Reaction 1 in Figure 8). This is by far the most common method used to form thiolated CTS, as summarized in Table 1, which records some examples of mercaptocarboxylic acids used, with L-cysteine (Cys), the tripeptide glutathione (GSH), and thioglycolic acid (TGA) being especially relevant. The final degree of functionalization (thiol group concentrations) on the thiomers are usually determined via Ellman’s reagent (5,5′-dithiobis-(2-nitrobenzoic acid), DTNB) according to literature protocols [109].

Some CTS derivatives with an enhanced hydrophilic character are often chosen, such as glycol CTS [26,110] and CTS-*graft*-PEG-SH, the latter being soluble in aqueous solutions with pHs above 8 [111]. These materials might show potential as auxiliary agents in oral drug delivery, where their solubility is likely beneficial. Similarly, the use of Cys as thiolating agent provides the material with an additional advantage: the presence of carboxylic groups increases the water-solubility of CTS at neutral and basic pHs. This characteristic is highly desirable, bearing in mind that the limited solubility of chitosan (i.e., at pH > 6.0) may compromise its application in various biomedical fields [62]. The *N*-acylation of CTS with fatty acids with diverse alkyl chain lengths and degrees of substitution modify the hydrophilic-hydrophobic balance of the material, and hence its properties for future applications. Thus, selected *N*-alkyl derivatives of CTS, although exhibiting reasonable water-solubility, can display self-assembly properties that lead to micellar aggregation, with potential applications as DDSs, nucleic acid transfection in gene therapy, and blood compatibility [112]. Thiolated CTS derivatives can self-assemble into core-shell structured NPs when CTS is thiolated using lipophilic ligands such as 4-mercaptobenzoic acid (4-MBA) [70]. On the other hand, a reduction in the degradation behavior of CTS-based thiomers is observed both when increasing the hydrophobicity of the thiol precursor, and when it is crosslinked through the formation of disulfide bonds, a useful feature in applications such as core-crosslinked NPs [25] and 3D bioscaffolds [62].

**Table 1:** Mercaptocarboxylic acids (HS-R-COOH) used for the preparation of CTS-based thiomers by means of 1-ethyl-3-(3-dimethylaminopropyl)carbodiimide (EDAC)-mediated coupling reactions (Reaction 1a in Figure 8).

Reaction Code	Compound		Ref.	Reaction Code	Compound		Ref.
	Formula	Abbrev.			Formula	Abbrev.	
**1a**	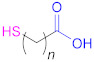 *n* = 1, 2, 3, 5, 7, 10 *n* = 1: Thioglycolic acid	*n* = 1: TGA *n* = 2: MPA *n* = 3: MBA *n* = 5: MHA *n* = 7: MOA *n* = 10: MUA	*n* = 1: [18,23,29,70] *n* = 2: [18,113] *n* = 3: [18] *n* = 5: [18] *n* = 7: [18] *n* = 10: [19,62]	**1a**	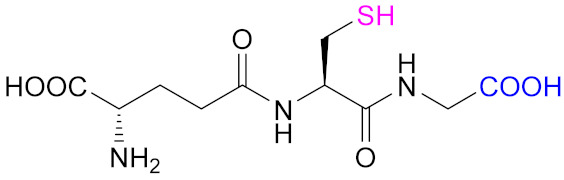 Reduced Glutathione	GSH	[26,70,110]
**1a**	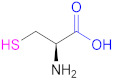 Cysteine	Cys	[62]	**1a**	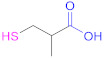 2-Methyl-3-sulfanyl propanoic acid	MSPA	[18]
**1a**	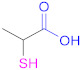 Thiolactic acid	TLA	[18]	**1a**	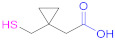 1-(1-(Mercaptomethyl) cyclopropane) acetic acid	MCA	[20]
**1a**	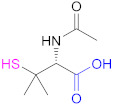 *N*-Acetylpenicillamine	NAP	[114]	**1a**	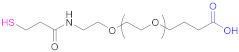 *O*-(3-Carboxypropyl)-*O*’-(2-(3-mercaptopropionyl amino)ethyl) polyethyleneglycol	--	[111]
**1a**	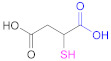 Mercaptosuccinic acid (Thiomalic acid)	--	[24]	**1a**	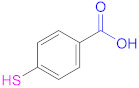 4-Mercaptobenzoic acid	4-MBA	[25,70]
**1a**	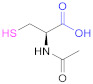 *N*-Acetylcysteine	NAC	[26,64,70,110,114]	**1a**	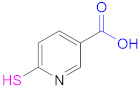 6-Mercaptonicotinic acid	6-MNA	[70,115]

Note: MBA: 4-mercaptobutanoic acid; MHA: 6-mercaptohexanoic acid; MOA: 8-mercaptooctanoic acid; MPA: 3-mercaptopropanoic acid; MUA: 11-mercaptoundecanoic acid.

The thiolation process can be improved by the combined use of *N*-hydroxysulfosuccinimide [20,110] or *N*-hydroxysulfosuccinimide sodium salt (Sulfo-NHS) with EDAC as a zero-length coupling agent (stabilization of intermediates formed) [19,62]. For example, being the single-EDAC thiolation procedure satisfactory for the coupling of ligands such as NAC and TGA, the GSH coupling reaction significantly improved the thiol yields when EDAC and NHS were added [70].

When using thiolating agents with unprotected free sulfhydryl groups such as the mercaptocarboxylic acids recorded in Table 1, disulfide linkages can be formed with the correlated decrease in free sulfhydryl group concentration in the polymer. If necessary, and in order to avoid it, a reducing agent can be added near the end of the coupling reaction, with tris(2-carboxyethyl)phosphine hydrochloride (TCEP) being one of the most widely used. For example, Croce et al. have applied this methodology to the development of a number of *N*-acyl thiomers as potential antibacterial materials [18]. Mueller et al. found that aromatic ligands such as 4-MBA and 6-mercaptonicotinic acid (6-MNA) required TCEP as a reducing agent in a final concentration of 10 mM [70]. The reduction process can take place after the coupling reaction and other reducing agents, such as DTT, can be used [20].

**Scheme 1:**
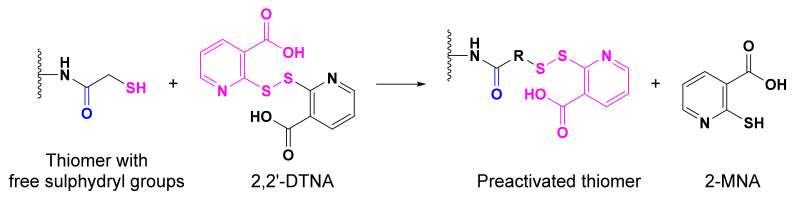
Example of preactivation reaction of thiomers containing free sulfhydryl groups with disulfides derived from nicotinic acid by thiol–disulfide exchange reaction (Reaction 1b in Figure 8). 2,2′-DTNA: 2,2′-dithiodinicotinic acid; 2-MNA: 2-mercaptonicotinic acid.

This is the reason why free sulfhydryl groups from freshly synthesized thiomers are frequently protected or/and preactivated to preserve the materials’ properties (Reaction 1b—preactivation—in Figure 8; Scheme 1). Among the protecting and preactivating groups, the (pyridin-2-yl)thio-based moiety not only has the ability to protect the mercapto group from oxidation reactions, but also preactivates it for reaction with Cys residues in the human body. In general, free unprotected thiol groups from thiomers can react with disulfide bonds of proteins, whereas *S*-protected/preactivated thiol groups from thiolated materials may react with free thiol groups from Cys residues in proteins [13]; also noticeable is the discovery that thiolated chitosan derivatives with thiols protected by a good leaving group are more mucoadhesive than their *S*-unprotected counterparts [116,117].

The protection and preactivation of sulfhydryl moieties in thiomers can take place by means of a thiol–disulfide exchange reaction. The disulfide molecules most frequently employed for this purpose are 2,2′- and 6,6′-dithiodinicotinic acid (2,2′-DTNA and 6,6′-DTNA, respectively) and 6,6′-dithiodinicotinic amide (6,6′-DTNAm), as recorded in Table 2. In addition, the dimer of 3-methyl-1-phenylpyrazole-5-thiol (MPPT) has been chosen with the same objective. The electron-withdrawing effect exerted by the π-deficient pyridine rings makes the synthesized asymmetric disulfide linkages more reactive to thiol–disulfide exchange reactions and more easily cleaved off, and hence, its preactivation is ensured [13].

This methodology was used, for example, to *S*-protect/preactivate quaternary ammonium CTS thiomers (QA-CTS-TGA-SH). The *S*-protected and preactivated polymer QA-CTS-TGA-S-6-MNAm was obtained by disulfide bond formation with 6-MNAm through reaction with 6,6′-DTNAm [116].

Nicotinic acid derivatives such as 2-MNA and 6-MNAm are preferred as leaving groups to 2-mercaptopyridine in these types of reactions due to safety reasons [118]. Therefore, the most used reagents to protect free thiol groups are 2,2′-DTDNA, 6,6′-DTNA, and 6,6′-DTNAm. Interestingly, the antimicrobial properties of MPPT were considered in the preactivation of CTS-TGA-SH with the dimer of MPPT by Müller et al. [83]. The preactivated CTS-S-S-MPPT released the antimicrobial MPPT into the medium, so this thiomer might be used for the treatment of infected tissues.

Quantification of the total free sulfhydryl groups remaining after the preactivation step indicated that although the reaction worked reasonably well, full protection of the sulphydryl groups was not achieved. For example, values close to 80% were found by Netsomboon et al. for EDTA functionalized CTS-Cys-2-MNA [48].

**Table 2:** Disulfides used for the preactivation via a thiol–disulfide exchange reaction of already-prepared thiomers (Reaction 1b in Figure 8).

Reaction Code	Compound		Ref.	Reaction Code	Compound		Ref.
	Formula	Abbrev.			Formula	Abbrev.	
**1b**	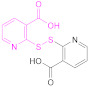 2,2′-Dithiodinicotinic acid	2,2′-DTNA	[26,48]	**1b**	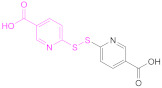 6,6′-Dithiodinicotinic acid	6,6′-DTNA	[92]
**1b**	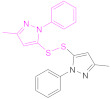 Dimer of 3-Methyl-1-phenylpyrazole-5-thiol	MPPT	[83]	**1b**	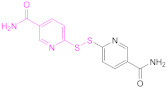 6,6′-Dithiodinicotiamide	6,6′-DTNAm	[22,116,119]

This drawback is overcome when protected mercaptocarboxylic acids (Table 3) synthesized “ex profeso” are used. They react (EDAC-mediated) with the amino groups from the polymer (Reaction 2 in Figure 8), so the global of the incorporated thiol groups is preactivated. Among the sulfur-containing reagents, 2,2′-DTDNA and NAC-6-MNAm stand out. The *S*-preactivated NAC-6-MNAm can be prepared via a thiol–disulphide exchange reaction between NAC and 6,6′-DTNAm [120].

#### 4.1.2. Reaction of Multiamino Polymers with Highly Reactive Carboxylic Acid Derivatives

##### Reaction with *N*-hydroxysuccinimide Esters

The use of activated carboxylic acid derivatives, such as reactive *N*-succinimidyl carboxylates, is necessary for the formation of an amide bond between the multiamino polymer and the reagent bearing the mercapto group without the concourse of a coupling reagent such as EDAC (Reaction 3 in Figure 8). These active esters usually carry the sulfhydryl groups conveniently masked to avoid interferences during either the coupling or the work-up reactions. They are usually protected as acetylthio- or 2-pyridyldithio groups (Table 4). The former can be cleaved by a basic medium such as sodium hydroxide, potassium carbonate, or sodium methoxide, or by treatment with hydroxylamine to render the free thiols [13,60,95,121]; in the case of 2-pyridyldithio groups, they will be deprotected either using a reductive reagent such as NaBH_4_ [93] or TCEP [55], or by a thiol–disulfide exchange reaction when treated with DTT, Cys, or GSH [12,122].

An interesting example that utilizes this methodology is the thiolation of glycol CTS polymers by treatment with sulfosuccinimidyl-6-(3′-[2-pyridyldithio]-propionamido)hexanoate (sulfo-LC-SPDP) and subsequent reduction with DTT [122,124]. The resultant thiomer was able to form stable NPs with anionic polymerized siRNA (poly-siRNA) through charge–charge interactions, resulting in loosely bound large structures. Consequently, the distance between the thiol groups decreased, which allowed undergoing intra- and intermolecular crosslinking by formation of disulfide bonds, leading to more condensed nanostructures.

The formation of the *N*-hydroxysuccinimide ester can also be achieved in situ by adding *N*-hydroxysuccinimide (NHS), as was the case with GSH [60]. Another alternative is the activation of the thiocarboxylic acid as *N*-hydroxybenzotriazole (HOBt) esters prior to its reaction with the multiamino polymer. Moreover, Liu et al. described the synthesis of preactivated NAC (Table 4) in organic solvents prior to its reaction with CTS at pH 5 [125].

This succinate ester methodology has also been applied to the functionalization of APIs such as the monoclonal antibody cetuximab (CTX). CTX was derivatized by reaction with *N*-succinimidyl-*S*-(acetyl) thioglycolate (SATA; Table 4), so that CTX would be anchored to poly(lactic-*co*-glycolic) acid (PLGA)-CTS NPs by means of a Michael reaction with the PEG extender [80]. Similarly, a methacrylamide-modified lysozyme containing a bioreducible linker was prepared by reaction with SATA. The antimicrobial enzyme was temporary immobilized in a dextran-based network, and then its release was triggered by GSH [123].

##### Reaction with Acetimidates

Imidoesters (or imidates) (R-O-C(=NH)-R’) react with nucleophiles such as amines to form amidines (Reaction 4 in Figure 8). The amidine functional group is among the most basic, noncharged ones. In general, they are more basic than amines. Their p*K*_a_ ranges between 5 and 12, so that they could be protonated at physiological pH (imidinium ions), making a polymer with low solubility, such as CTS, soluble at physiological pH [95].

The imidoester reaction scheme for the chemical modification of chitosan was used for the first time by Kafedjiiski et al. in 2005 [121]. They used isopropyl (acetylthio)acetimidate (*i*-PATAI) as the thiol-bearing chemical with its sulfhydryl group deprotected as the *S*-acetyl group (Table 4), and it was observed that the deprotection of *S*-acetyl groups could partially and spontaneously occur at pH ≈ 7.0 [121]. To achieve a quantitative deprotection, treatment with NaOH or NH_2_OH should be conducted, the latter being a method used at near neutral pH. In general terms, imidoesters react rapidly with amines (1.5 h) under the optimal conditions (30 °C and pH 7–10), in contrast with the reaction with 2-iminothiolane, which ends after 24 h and shows unintended cyclization side reactions [126]. Since CTS is not soluble at such pH, the reactions are conducted at pH 6.5–7.0 [121]. However, disulfide bond formation occurs. When CTS reacted with *i*-PATAI, and then deprotected with NH_2_OH, the 48% of immobilized thiol groups were participating on disulfide linkages [95]. The formation of disulfide linkage is directly correlated with thiolate concentration, so the higher the pH is, the greater the ratio of sulfhydryl groups involved in disulfide bond formation becomes. For example, films of CTS–NAC conjugates were crosslinked at basic pH (pH 11) with a substantial improvement in their mechanical properties, and with almost no mercapto groups present in the materials [64]. This is the reason why an additional treatment with a reducing agent is advisable in this methodology, with TCEP being a common choice [95].

#### 4.1.3. Thiolation in Two Steps: Nucleophilic Reactions of Multiamino Polymers with Chloro-Derivatives and Incorporation of Thiol Moiety

Other synthetic options include the use of bis-electrophiles that can act as anchoring moieties between the multiamino polymer and a thiol-bearing nucleophile. Chloroacetyl chloride and epichlorohydrin are the two bis-electrophiles most widely used in the derivatization of CTS.

##### Reaction with Chloroacetyl Chloride

Acyl chlorides are among the most reactive carboxylate derivatives, and they can react readily with amino groups. Chloroacetyl chloride is a double electrophile that can ensure the connection with two nucleophiles, such as the amino groups from the polymer and a nucleophile from the thiol carrier. The reaction of CTS with chloroacetyl chloride in the presence of pyridine leads to the formation of an amide bond (Reaction 5 in Figure 8). The as-synthesized chlorinated chitosan was further transformed in the thiol structure via thiourea [51]. The thiouronium salt intermediate is usually cleaved by treatment with sodium hydroxide [49,51,116]. Due to the high reactivity of acyl chlorides, the reactions are not usually restricted to amino groups. In polysaccharides and under the reaction conditions, a certain degree of their hydroxyl groups may react with chloroacetyl chloride, and hence the degree of thiolation per monosaccharide unit can be higher than one in the final polymer.

##### Reaction with Epichlorohydrin

Similar to what was found with chloroacetyl chloride, this doubly electrophilic reagent can react with both amino groups and hydroxyl moieties activated in basic media. This feature is related to some crosslinking phenomena found by this methodology, and therefore, this approach is frequently chosen when looking for the preparation of resins, beads, or microspheres, especially for the adsorption of heavy-metal cations such as Au(III), Pd(II), Pt(II), Hg(II), Cu(II), and Ni(II) [50,52,127]. Once the oxirane ring is opened by the nucleophilic attack of the amino or alkoxide groups, the incorporation of the thiol moiety can take place via treatment with thiourea [49,52] or via reaction with selected thio-functionalized triazoles [127] or thiazoles [50] (Table 5) rendering the thiolated material (Reaction 6 in Figure 8). Contrary to the cases studied above, the joining bond between the multiamino polymer and the thiol-bearing residue is a basic secondary amino group that, from the toxicological point of view, might be disadvantageous for their use in humans [13].

One example of this methodology is the preparation of magnetic CTS microspheres as a chelating agent. Magnetic CTS microspheres, previously crosslinked with glutaraldehyde, were the starting material for the grafting of sulfur groups using epichlorohydrin as a second crosslinking agent. The magnetic CTS microspheres were chemically modified with thiourea for adsorption of metal ions (Hg^2+^,Cu^2+^, and Ni^2+^) [52].

#### 4.1.4. Ring Opening of Reactive Thiolating Cycles: 2-Iminothiolane and Thiolactones

One of the most reactive thiolating cycles is the 2-iminothiolane (Traut’s reagent; Table 6), a well-known reagent frequently used in the immobilization of thiol groups to primary amino groups of proteins [69]. This cycle reacts with primary amines to introduce sulfhydryl groups while maintaining charge properties like the original amino group. The reaction of 2-iminothiolane with CTS renders CTS-(4-thiobutylamidine) conjugates (Reaction 7 in Figure 8). This coupling reaction has been tested at acidic (pH 5.0, 6.0), neutral (pH 7.0) and basic (pH 10.0) conditions [30,69,70,128]. Due to the lower reactivity of 2-iminothiolane in acidic media [128], the degrees of thiol substitution found at neutral pH were substantially higher than those achieved under equivalent conditions, but at acidic pH [30,69]. The use of basic pH did not render the expected enhanced degree of thiol substitution, probably due to the reduced solubility of CTS in such media [69]. On the other hand, and as mentioned before, the higher the pH of the medium, the greater the tendency of disulfide bond formation from free thiol groups; therefore, a compromise solution should be reached regarding the pH of the reaction media. Additionally, the reaction of CTS with 2-iminothiolane is usually conducted under an inert atmosphere to prevent oxygen-mediated thiol oxidation [25,67], and the addition of sulfhydryl-based reagents such as DTT and 2-mercaptoethanol has also been reported with the aim of keeping the thiol groups as such [30,69].

Another type of reactive cycles used as thiolating agents are the thiolactones (Reaction 8 in Figure 8), with the most widely used being those derived from γ-thiobutyrolactone, with or without a substituent on the γ-position of the cycle (Table 6). As in any nucleophilic reaction in which amino groups are involved, the pH of the media is crucial to optimize the degree of thiol substitution. These reactions are conducted at pH 6–7 in every case using different buffer systems, such as the 2-(*N*-morpholino)ethanesulfonic acid (MES) [67,129], classified as a “Good’s buffer”, or imidazole buffer [130]. The reaction of multiamino polymers with such cyclic thiolating agents leads to polymer-(4-thiobutylamide) conjugates in good yields without the concomitant release of small molecules found in other methods.

To sum up, a plethora of synthetic methods is available for the thiolation of CTS, the most popular being the coupling reaction with mercaptocarboxylic acids (protected/preactivated or not) and the reaction with reactive carboxylic acid derivatives such as *N*-hydroxysuccinimide esters. These procedures can be implemented to achieve the derivatization of other multiamino polymers, with the consequent enlargement of potentially available thiolated materials with tailor-made properties.

**Table 6:** Thiolactones and 2-iminothiolane used as thiolating agents in reactions with multiamino polymers (Reactions 7 and 8 in Figure 8).

Reaction Code	Compound		Ref.	Reaction Code	Compound		Ref.
	Formula	Abbrev.			Formula	Abbrev.	
**7**	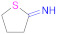 2-Iminothiolane	--	[30,69,70]	**8**	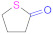 γ-Thiobutyrolactone	--	[53]
**8**	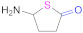 Homocysteine thiolactone	HT	[130]	**8**	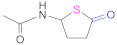 *N*-Acetylhomocysteine thiolactone	--	[67,129]

### 4.2. Strategies for the Thiolation of Multicarboxyl Polymers

Another interesting polymer for biomedical and pharmaceutical applications in tissue engineering, regenerative medicine, and as a drug delivery system, is the anionic biopolymer hyaluronic acid [68]. HA is a nontoxic, biocompatible, greatly biodegradable, and nonimmunogenic polysaccharide that is present in connective tissue as a major component of the extracellular matrix, and can be metabolized via enzymatic hydrolysis by hyaluronidase (HAase). It is a material that can also induce cell proliferation, wound healing, and angiogenesis [78,131,132]. Perhaps the most remarkable advantage of HA is its potential for active targeting without any additional targeting ligands. HA has a strong affinity for cell-specific surface markers such as CD44 and RHAMM, and as a result, has drawn much attention from the scientific community [132].

HA is constituted by alternating disaccharide units of D-glucuronic acid and *N*-acetyl-D-glucosamine, linked via alternating β-(1→4) and β-(1→3) glycosidic bonds. Due to the presence of carboxylic groups (-COOH) and hydroxyl groups (-OH), HA can be chemically modified by robust synthetic strategies, and such is the case for the thiolation methods. The attachment of free thiol groups on the polymeric backbone provides the new materials with enhanced mucoadhesive features, as well as in situ gelling properties [17,133]. Although some commercial HA-derived thiols are available and have been utilized in various research works [134,135], there are reliable thiolating approaches than can be applied to a number of multicarboxyl polymers: from the natural HA [14] and pectin (Pec) [29] to the synthetic PAA and poly(methacrylic acid) (PMAA), and their copolymers [85].

**Figure 9:**
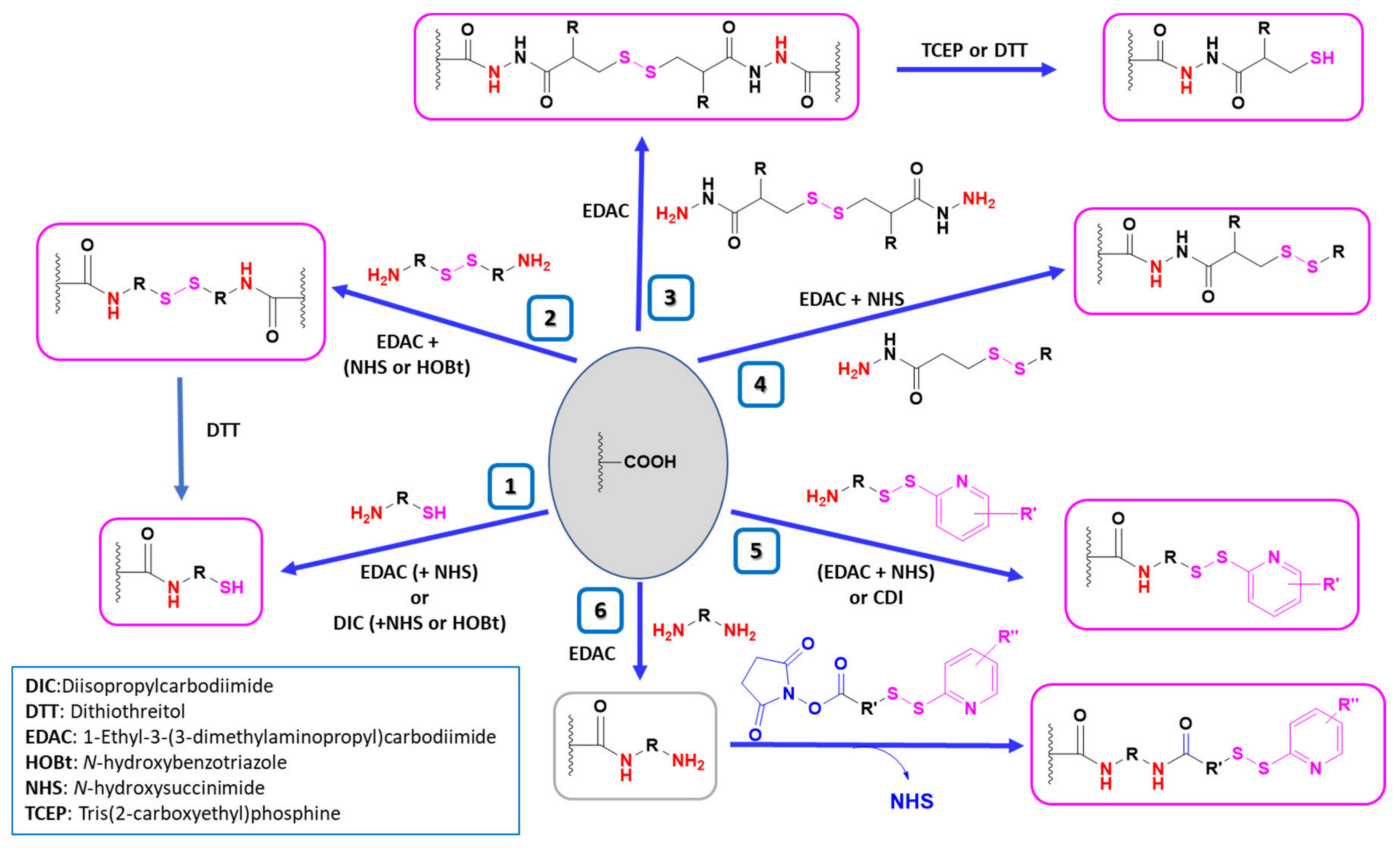
Reported routes to thiolate multicarboxyl polymers.

The thiolating methods designed for multicarboxyl polymers are mainly based on EDAC-mediated coupling reactions (Reactions 1–6 in Figure 9), already described in the previous section. The thiolating reagents can be aminothiols or their dimers (Reactions 1 and 2 in Figure 9), such as cysteamine (CSA), Cys and cystamine (Table 7), preactivated aminothiols (Reaction 5 in Figure 9), and symmetric or asymmetric dithiobis(dihydrazide)s (Reactions 3 and 4 in Figure 9).

The coupling reaction between an anionic polymer and a free, dimerized, or preactivated aminothiol (Reactions 1, 2, and 5, respectively, in Figure 9) is usually conducted in aqueous media, with EDAC being the carbodiimide of choice. To potentiate the reactivity of the carboxylic acid groups of the anionic polymer before coupling occurs, the addition of NHS [15,16,71,78] or HOBt [31,136] is quite common. On the other hand, Baus et al. studied the impact of the absence of water during the synthesis of thiolated PAA both from Cys and Cys-2-MNA [85]. They found that the extent of functionalization under aqueous conditions was lower than with organic solvents. The approach of a water-free thiomer synthesis improved immobilization of thiol groups up to 4.2-fold in comparison to functionalization in aqueous media, probably due to the greater stability of the coupling reagents used (*N*,*N*′-diisopropylcarbodiimide (DIC) and 1,1′-carbonyldiimidazole (CDI) for Cys and Cys-2-MNA, respectively) and the active intermediates involved. When cystamine, the dimer of cysteamine (CSA) is used as a thiolating agent, the obtained crosslinked materials have to be reduced, usually with the concourse of DTT [15,31,136].

Other very popular thiolating reagents when functionalizing HA are dithio(bis)hydrazides such as *N*-acetyl-*S*-((3-hydrazineyl-3-oxopropyl)thio)-cysteine (NAC-TPH) [17], 3,3′-dithiobis(propanoic hydrazide) (DTPH) [14,63,79,137], and its derivatives (Table 7; Reactions 3 and 4 in Figure 9) [63]. Similar to what occurs in the synthetic pathway with dimers of aminothiols, the presence of disulfide linkage in the hydrazide may necessitate a reduction step after the coupling reaction [14,63,79,137].

**Table 7:** Methodology (related to Figure 9), anionic polymer, thiolating agents, and coupling reagents used for the preparation of thiomers derived from multicarboxyl polymers.

Reaction Code	Polymer	Compound		Coupling Reagents Used and Other Reaction Details	Ref.
		Formula	Abbrev.		
**1**	HA	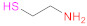 Cysteamine	CSA	1.- EDAC + NHS (2 h) 2.- NH_2_-R-SH	[16,78]
**1**	PAA	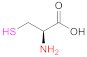 Cysteine	Cys	EDAC (in H_2_O) or DIC + (NHS or HOBt) (in DMF-DCM mixtures)	[85]
**1**	Pec	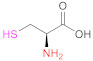 Cysteine	Cys	EDAC	[29,71]
**2**	HA	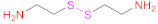 Cystamine		1.- EDAC + NHS 2.- DTT	[15]
**2**	HA	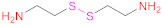 Cystamine		1.- EDAC + HOBt 2.- DTT	[31,136]
**3**	HA	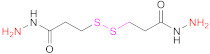 3,3’-Dithiobis(propanoic hydrazide)	DTPH	1. EDAC 2. TCEP	[14,79]
**3**	HA	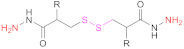 **R: H**: 3,3’-Dithiobis(propanoic hydrazide) **R: NHAc**: 3,3’-Dithiobis(2-acetamidopropanoic hydrazide) **R: NH_2_ HCl**: 3,3’-Dithiobis(2-aminopropanoic hydrazide)	**R:H**: DTPH	1. EDAC 2. DTT	[63,137]
**4**	HA	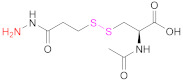 *N*-acetyl-*S*-[(3-hydrazineyl-3-oxopropyl)thio]-cysteine	NAC-TPH	EDAC + NHS	[17]
**5**	PAA	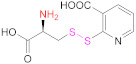 Cysteine 2-mercaptonicotinic acid adduct	Cys-2-MNA	EDAC + NHS (in H_2_O) or CDI (in DMF-DCM mixtures)	[85]
**5**	Pec	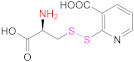 Cysteine 2-mercaptonicotinic acid adduct	Cys-2-MNA	EDAC + NHS	[71]
**6**	HA	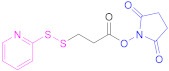 *N*-Succinimidyl 3-(2-pyridyldithio)propionate	SPDP	1.- Diamine(C6) + EDAC 2.- SPDP 3.- SH-PEG-SH	[138]

Note: HA: hyaluronic acid; CDI: 1,1’-carbonyldiimidazole; DCM: dichloromethane; DIC: 1,3-diisopropylcarbodiimide; DMAP: 4-(dimethylamino)pyridine; DMF: *N*,*N*-dimethylformamide; DTT: dithiothreitol; EDAC: 1-ethyl-3-(3-dimethylaminopropyl)carbodiimide; HOBt: *N*-hydroxybenzotriazole*;* NHS: *N*-hydroxysuccinimide*;* PAA: poly(acrylic acid); Pec: pectin; TCEP: tris(2-carboxyethyl)phosphine; XrL: crosslinked.

**Scheme 2:**
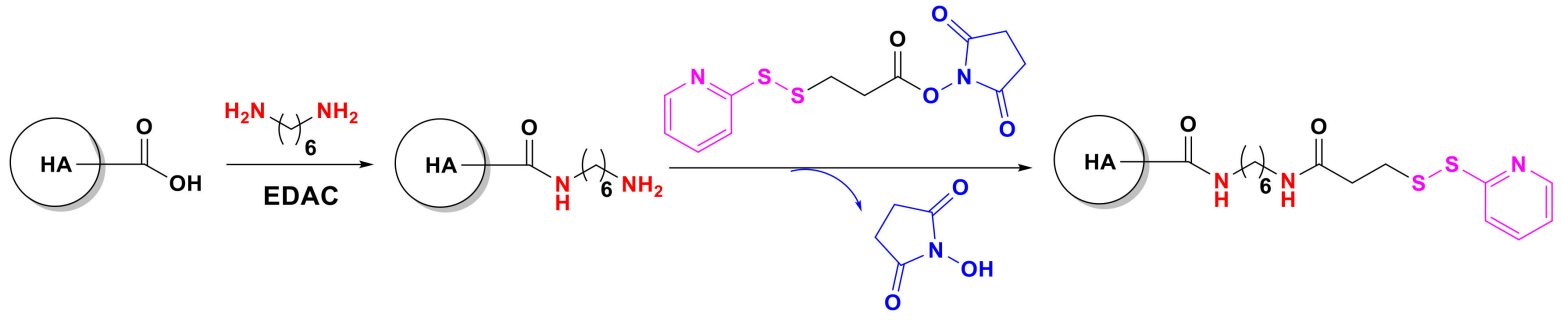
Transformation of HA to preactivated thiol-bearing HA (Reaction 6 from Figure 9) for its later crosslinking with the macro-crosslinker PEG-dithiol [138].

Other approaches rely on the initial coupling reaction between the carboxylic acid groups from anionic macromolecules and one of the amino groups from diamines to render multiamino polymers (Reaction 6 in Figure 9). These materials can then be thiolated by any of the methods listed in Figure 8. This is the case of the preparation of injectable HA hydrogels crosslinked with the macro-crosslinker PEG-dithiol reported by Choh et al. [138]. HA was coupled with diaminohexane using carbodiimide chemistry to yield amino-derivatized HA (HA-CO-(CH_2_)_6_-NH_2_), followed by a reaction with SPDP, generating HA derived with pyridyl disulfide, ready for the crosslinking process (Scheme 2).

In summary, interesting and reliable methods are available for preparing HA-based thiomers with smart properties for their applications in cancer therapy.

### 4.3. Nanostructures from Chitosan and Hyaluronic Acid-Based Thiomers

In the design of NPs as DDSs, particle size is an essential parameter for cellular uptake. For example, the permeability of the NPs through the intestinal mucosa decreases when the particle size reaches 400–500 nm [139]. On the other hand, NP-based delivery systems with size ranges of 100–500 nm are known to efficiently accumulate at tumor sites through fenestrate vasculature (EPR effect) [140]. Therefore, when preparing nanostructured DDSs from polysaccharides such as CTS or HA, the molecular weight of the material exerts a significant effect on formulation size and drug delivery. This outcome was demonstrated for CTS glutamate and CTS aspartate NPs produced by ionotropic gelation with sodium tripolyphosphate (TPP), using bovine serum albumin as a model protein. In those experiments, CTS from low-molecular-weight (35 kDa) to medium- and high-Mw CTS (100 and 800 kDa) were used, and the particle sizes varied from 195 to 3450 nm. Nanoparticles prepared with the highest molecular weight CTS showed the largest sizes [141]. This is the reason why low-weight CTS is usually chosen in the design of nanoparticulated DDSs; for example, NPs prepared for nose to brain codelivery of alpha-cyano-4-hydroxycinnamic acid and the monoclonal antibody cetuximab were obtained by ionic interaction between oligomeric CTS and poly(lactic-*co*-glycolic) acid (PLGA) [80].

This effect was also studied in docetaxel-loaded NP prepared from amphiphilic CTS–GSH–*graft*–PMMA (poly(methyl methacylate)) thiomers. The starting CTS polymers had molecular weights of 20, 50, and 100 kDa (Table 8) [27]. The NPs were drawn up by radical emulsion polymerization of MMA on thiolated CTS–GSH, using cerium IV ammonium nitrate (CAN) as a radical initiator. The amphiphilic *block*-copolymers were capable of self-assembling in aqueous media. The mean sizes of the NPs (ranging from 153 to 264 nm) were highly and positively dependent on the molecular weights of thiolated CTS used. Thus, the thiolated CTS with the lowest molecular weight (20 kDa) and the highest free thiol content on their surface were used for the optimal formulation of DTX-loaded NPs [27]. Self-assembly of CTS-based thiomers with hydrophobic subdomains was also observed for the amphiphilic carboxymethyl-CTS derivatized with 4-mercaptobenzoic acid (4-MBA) via EDAC procedure. These NPs were core crosslinked by air oxidation under ultrasonication. Adjacent thiol groups in the hydrophobic core produced disulfide bonds, leading to pH- and redox-responsive NPs with average diameter of 160 nm [25].

One of the most widely used methods for NP formation from ionic polysaccharides such as HA and CTS-based thiomers is ionic gelation (ionic crosslinking) mediated by a counter ion. Thus, CTS-based NPs were formulated via gelation with sodium sulfate as nanocarriers for doxorubicin (DOX) and antisense oligonucleotide (ASOND). They displayed a spherical, uniform shape with a mean particle size of 150–300 nm [114]. Another option for CTS-based particles is ionic gelation with TPP, feasible for both CTS and thiolated CTS–GSH [29]. For anionic thiomers such as HA or PAA, cations like Ca^2+^ can be used to stabilize the NPs. Dünnhaupt et al. achieved the successfully load of the hydrophilic macromolecule fluorescein isothiocyanate–dextran (FD4) in NPs of PAA–Cys stabilized with Ca^2+^ for its oral delivery [29]. They also found that the addition of trehalose in a concentration of 3% to NP suspensions avoided particle aggregation.

A general approach to produce NPs is by polyelectrolyte complex formation between two ionized polymeric materials with opposite charges in their structures. For example, NPs based on thiomers from quaternary ammonium (QA) CTS (both QA-CTS-TGA-SH and the preactivated thiomer QA-CTS-TGA-S-S-6-MNAm) were prepared by self-assembly upon addition of reduced-molecular-weight hyaluronic acid (rHA: 470 kDa), and displayed average size of 370 nm. They were tested as oral DDSs, using the model compound FD4. It was found that when the drug was encapsulated in the more mucoadhesive NP type (the preactivated ones), it exhibited higher oral bioavailability than that enclosed in the less mucoadhesive ones [116]. Another example for this methodology is the preparation of a surface-modified nanostructured lipid carrier (NLC). First, the preparation of curcumin-loaded NLC was conducted by the melt emulsification technique, leading to a negatively charged NLC. They were later surface-modified by polyelectrolyte complex interaction with the thiomer CTS–NAC, leading to the ophthalmic coated formulation [125].

This method can be extended to the design of ionic complexes in which three different macromolecules are involved: positively charged polymetformin-based NPs (loaded with DOX), the negatively charged plasmid-encoding IL-12 gene, and a thiolated HA, as will be discussed below [136].

The stability of the nanocomplexes formed by oppositely charged macromolecules can be ensured by the appliance of a bonus intraparticle interaction; for example, by means of the addition of small ions such as TPP, or the formation of disulfide bridges. Thus, nanosuspensions loaded with the antituberculosis drug isoniazid were obtained by polyelectrolyte complex formation between thiolated CTS–TGA and HA, using sodium-tripolyphosphate (TPP) as an extra crosslinker [23]. This methodology is equivalent to that reported for the development of polyelectrolyte complexes of thiomalyl CTS and insulin for the oral delivery of the latter, in which TPP played a similar role [24].

Taking advantage of the use of thiolated polymers, the stabilization of polyelectrolyte complexes can be boosted by the formation of disulfide linkages, generally exerted by air-mediated oxidation of thiol groups at neutral or slightly basic pHs. An example for this approach is the formulation of a stable polyelectrolyte complex of carboxymethyl(sulfate)-CTS-4-thiobutylamidine and the basic fibroblast growth factor (dFGF). At pH 7.4, in the presence of oxygen, the thiol groups from the CTS-based thiomer were easily oxidized to disulfide bonds, adding stability to the NPs by forming an in situ crosslinked structure [30]. Similarly, thiolated glycol CTS can form stable nanoparticles with poly-siRNA through charge–charge interactions and self-crosslink via disulfide bond formation after incubation in HEPES buffer (pH 8.0) for 1 h at 37 °C. This structure provided sufficient stability from nucleases while still allowing for mono-siRNA release in the cytosol upon systemic delivery of poly-siRNA [122,124].

A triple interaction has been reported in NPs prepared from HA–SH. The thiomer formed a polyelectrolyte complex poly-siRNA, which was stabilized by the addition of calcium phosphate and disulfide bonds produced by thiol oxidation [31].

To conclude, HA- and CTS-based thiomer NPs are capable of encapsulating small and biomacromolecular APIs involved in anticancer therapy with efficiency, mainly by means of ionic gelation and polyelectrolyte complex formation. These two strategies can be combined with each other and with disulfide bond formation to lead to stable and reduction responsive drug-loaded NPs.

## 5. Thiomer Conjugates in Anticancer Therapy

### 5.1. Thiomer-Based Conjugates for Taxanes and Other Anticancer API Administration

**Figure 10:**
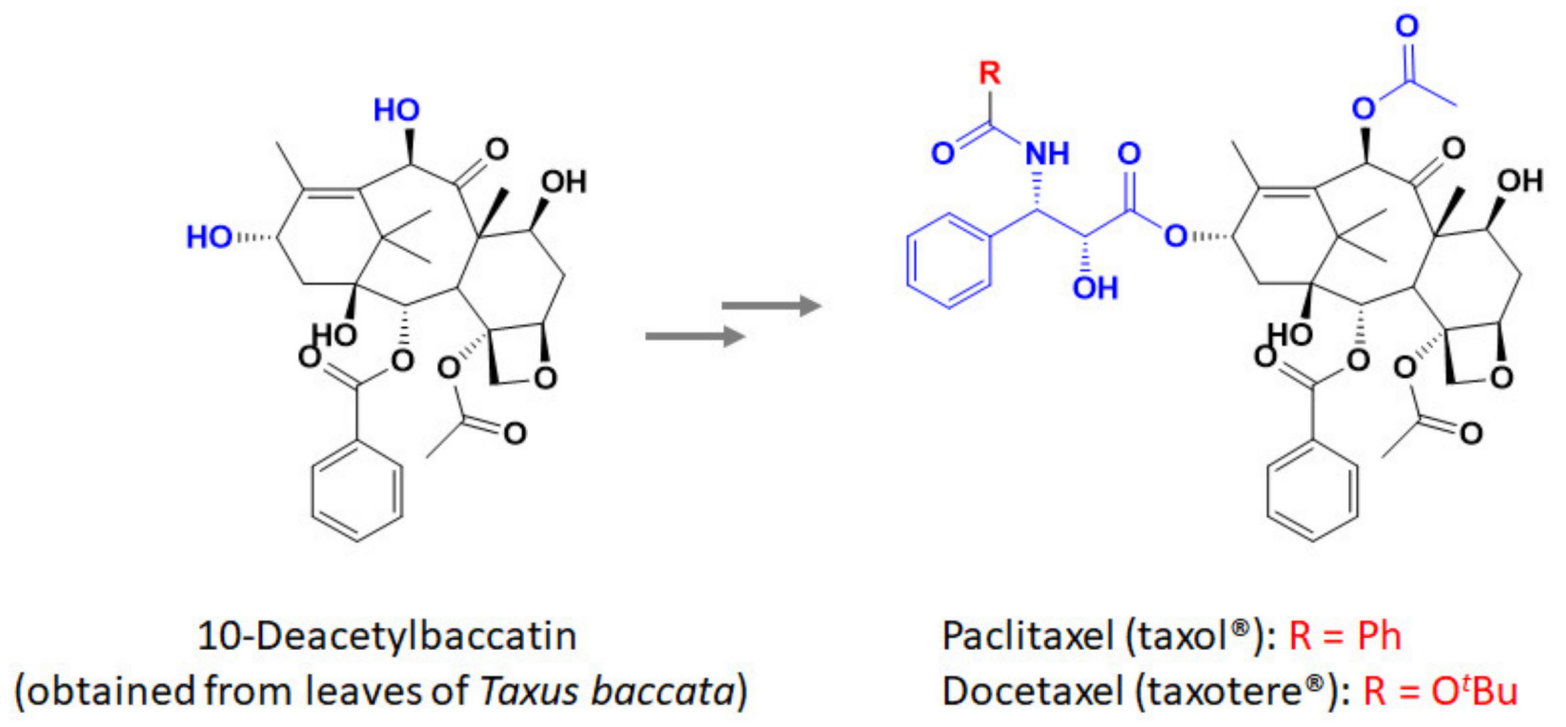
Chemical structures of paclitaxel, docetaxel, and 10-deacetylbaccatin, the building block for their semisynthesis.

Taxanes are common chemotherapeutic agents used for the treatment of cancers that form a solid discrete mass (known as solid tumors), such as prostate or breast cancer. They are obtained by processing the bark of some taxa of yew trees; for instance, the most clinically relevant taxanes, which are paclitaxel (PTX, commercialized under the brand Taxol^®^) and docetaxel (DTX, sold under the brand Taxotere^®^), can be isolated when harvesting the bark of *Taxus brevifolia* (Pacific yew tree) [37] and that of *Taxus baccata* (European yew tree) [27], respectively (Figure 10). Nonetheless, today the obtention of these molecules has been refined by the development of semisynthetic or even totally synthetic chemical routes of production, such as the one described by Baloglu et al. for paclitaxel from baccatin III [142]. This is very relevant for the development of these drugs in the clinical field, considering that large-scale extraction of ingredients straight from the forest may be very detrimental to the environment [142]. In this sense, it is obvious that any substance that can be obtained by greener and more environmentally friendly methods will more probably succeed in the long run (due to more availability, less economical costs, better preservation of the environment, etc.) than any other that can only be isolated from natural resources.

**Figure 11:**
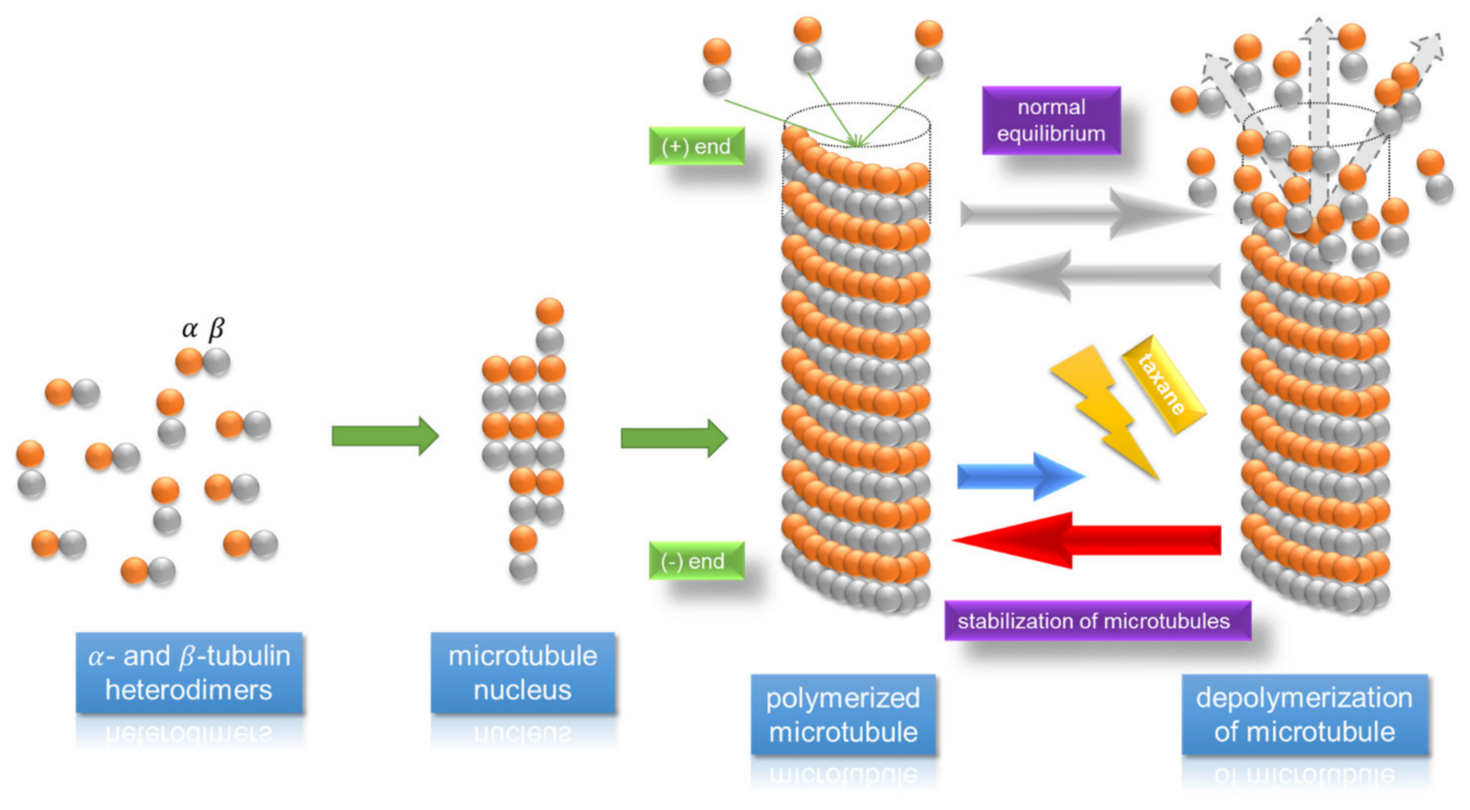
Mechanism of action of taxanes. Paclitaxel and docetaxel can bind microtubules during the M phase of the cell cycle, therefore blocking their depolymerization. By doing so, they interfere with the function of the spindle apparatus, so chromosomes are unable to move to the opposite sides of the dividing cells structures during anaphase.

The use of these compounds in hematopoietic and lymphoid malignancies has not been fully exploited and is not very widespread yet, remaining in the background compared to other antineoplastic substances that exert their cytotoxicity through a very similar mechanism, such as vincristine or vinblastine. Such mechanism of action is based on the inhibition of microtubular depolymerization [27], with which the compound manages to stop the division of tumor cells during the mitosis (M phase) of the cell cycle [143], as shown in Figure 11. This is because the mitotic spindle (made of microtubules), and more specifically microtubule reconfiguration, is essential during chromosome segregation. Therefore, by inducing its failure, cancer cells undergo mitotic catastrophe and perish [144].

Even though PTX has been reported to be effective when used in tumors previously characterized as refractory to conventional chemotherapy [145], some studies show that DTX has about twice the cytotoxic effect of paclitaxel on tumor cells [146]. In any case, both substances have demonstrated to be very promising among those found in the pharmacopoeia of anticancer therapy. However, they are usually associated with serious adverse effects when administered to patients, generally as injectable dosages [147]. For example, paclitaxel needs to be mixed with Cremophor EL^®^ (polyoxyl 35 hydrogenated castor oil, a rather toxic molecule) and anhydrous ethanol in order to enhance its solubility to an adequate level for intravenous administration [37]. As can be expected, this complex formulation does not work as an inert vehicle; in fact, it triggers a wide range of biological effects, some of which have important clinical implications, including severe anaphylactoid hypersensitivity reactions, hyperlipidemia, abnormal lipoprotein patterns, aggregation of erythrocytes, and peripheral neuropathy [147]. Other studies have shown that it can also cause neurotoxicity, nephrotoxicity, and alterations of the endothelium and the vascular muscles, leading to vasodilatation, labored breathing, lethargy, and hypotension [148].

Moreover, these microtubule-targeting agents also present limited oral bioavailability, which is an aspect that restricts their clinical use in cancer treatment [149]. This is a major issue to be solved, considering their potential as a chemotherapeutic agent. Thus, some groups have been investigating about the possibility of protecting these taxanes in novel formulations that could allow oral administration [13,27,37,94,148,150,151]. This might solve some of the problems associated with the parenteral injection of paclitaxel or docetaxel, probably erasing the need of mixing it with such toxic surfactants, thus reducing their systemic adverse effects.

One of these new approaches involves the administration of taxanes via such NPs as, for example, those with a core made of PMMA and coated with thiolated CTS [37]. This formulation has been tested for both paclitaxel [37] and docetaxel [27] on colon and breast cancer cells. The chitosan used in this study, which was subsequently thiolated, was low-molecular-weight chitosan, obtained by prior degradation of commercial medium-molecular-weight chitosan with NaNO_2_ [27,37]. The use of thiolated chitosan coatings, compared to chitosan-based ones, has been proved to be very beneficial to the oral administration of these taxane-loaded NPs, since the functionalization of the polymer with thiol groups has ameliorated mucoadhesiveness up to 140 times [150]. In fact, thiolation of biopolymers is, so far, one the most widely used strategies to enhance mucoadhesion [133]. This is essential when considering oral administration, because one of the main goals for the drug-loaded NPs is to remain anchored to the mucus layer as much time as possible, from which the taxanes will be taken up by the epithelial cells eventually. Free thiol groups from the thiomer will not only interact with the cysteine-rich subdomains found in mucins [152] (facilitating mucoadhesion), but also among the thiomer chains themselves. The latter will result in the formation of disulfide bonds inside the coating of the NPs, which has been proved to occur in a time-dependent and controllable manner [13]. In a nutshell, a highly cohesive polymer network can be produced thanks to this stable crosslinking [13], which serves as extra protection when administering the loaded compound. Apart from this, the fact that CTS is a cationic polymer (zeta potentials of the NPs were proved to be positive [27,37]) adds to the attaching forces participating to mucoadhesiveness; in this case, electrostatic interactions will also have a role in anchoring the NPs to the mucus layer of the intestine due to the anionic nature of the mucus.

Furthermore, the CTS coating can act as a chelant agent for metallic bivalent ions needed for some intestinal enzymes to function, such as aminopeptidase N and chymotrypsin, which can be involved in the deactivating of the therapeutics molecules [13,27]. Thus, by blocking the degrading activity of these enzymes, the successful intestinal uptake for the taxanes will be boosted, and hence, their half-life increased when administered orally. Finally, another relevant aspect that should be highlighted is the improved permeation of drugs through the gastrointestinal tract in the presence of thiolated polymers. Thiomers have demonstrated their ability to open tight junctions between epithelial cells by means of their thiol moieties, groups that are capable of inhibiting the protein tyrosine phosphatase, responsible for the closing process of such tight junctions [94]. Therefore, the access of the drugs to the systemic circulation will be promoted, with a concomitant improvement in the bioavailability of the therapeutic agent, thus it makes sense to refer to these materials as permeation enhancers. This process is known to be mediated by glutathione [94].

The studies that we have referenced in this section demonstrate the obvious potential that CTS-coated NPs have in the biomedical field. Focusing now on the pharmacokinetics and pharmacodynamics of these formulations, the studies that investigated them gave rather clear proof of high encapsulation efficiency of the nanoparticles (~90% for DTX [27] and ~98% at maximum for paclitaxel [37]), sustained released of the drug after a burst at the beginning [27,37], increased transportation of the drug and great cellular uptake [27,37], selective cytotoxic effect in cancer cells [27,37], biodegradability, and biocompatibility.

Other possibilities have also been tackled regarding the use of CTS as part of taxane-loaded NPs, which serve as carriers for the drug in oral administration. One study approached this issue by creating NPs made of the random copolymer poly(lactide-*co*-ε-caprolactone)-d-α-tocopheryl polyethylene glycol 1000 succinate (PLA–PCL–TPGS) and then modified superficially with thiolated chitosan [151]. These were shown to be potentially successful for the delivery of docetaxel to lung cancer cells [151]. PLA–PCL was used to strengthen the polymer, while TPGS was intended to make the loaded compound more permeable though biological membranes [151]; in fact, previous studies showed that this derivative of vitamin E can enhance absorption of drugs by inhibiting the efflux pumps in cancer cells (such as P-gp) while being nontoxic to healthy ones [153,154].

Just as the ones presented before, this study concluded with the same ideas: the NPs showed good cellular uptake and drug absorption (best results when they were modified with thiolated chitosan), sustained released, low systemic toxicity, biocompatibility, biodegradability, protection from drug degradation in gut, reduction of multidrug resistance (via blocking of P-gp), high encapsulation efficiency, and overall effectiveness [151]. Moreover, this work introduced a very interesting idea: thiolated chitosan per se has an antitumor effect as well. It was observed that caspase-3-dependet apoptosis was induced in lung cells when exposed to chitosan [151]. Therefore, this biopolymer itself may be acting synergistically with the loaded anticancer agents, which gives it another advantage, now not just working as a plain carrier but also contributing to cancer cell mortality.

**Figure 12:**
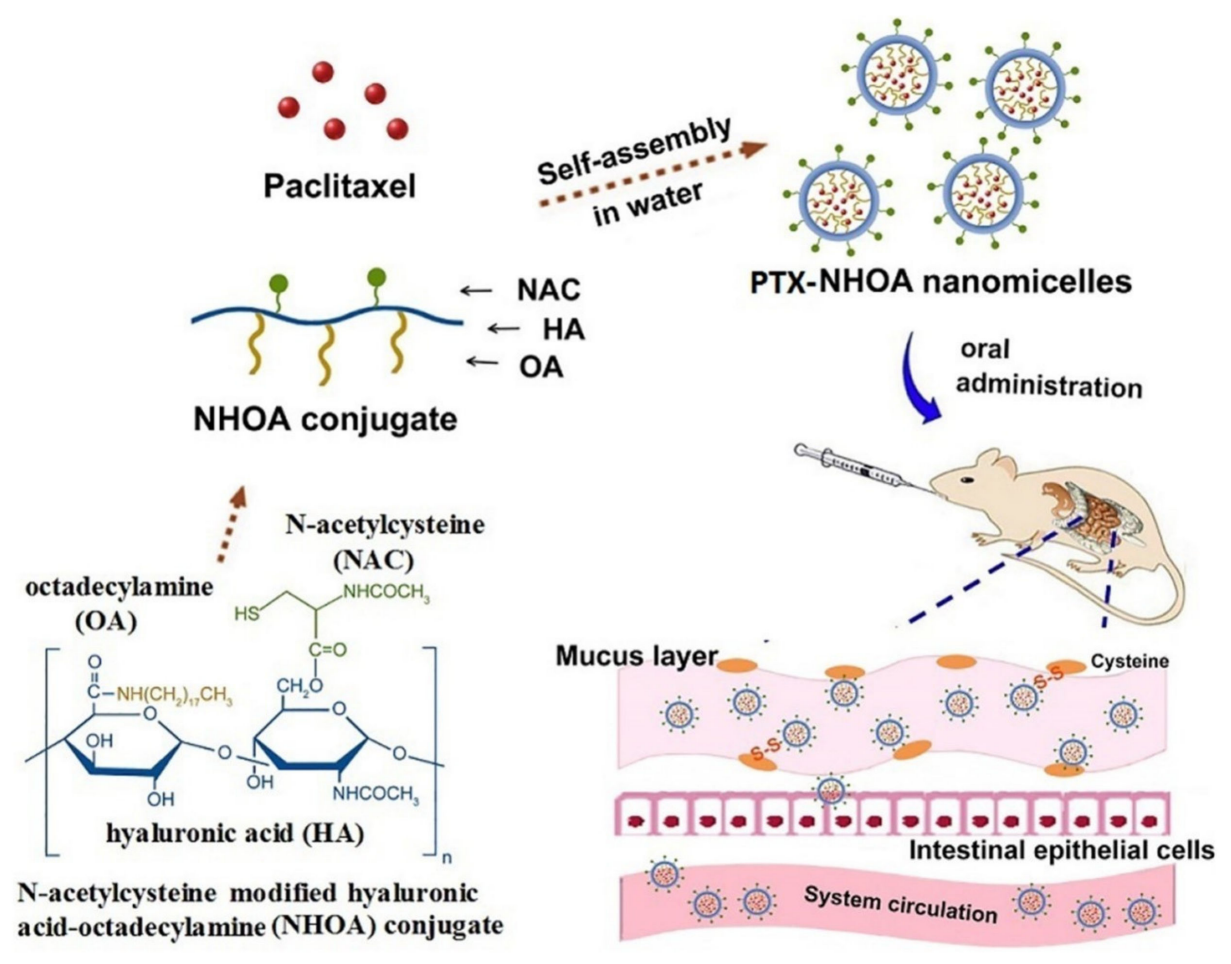
Summary of the assembly of PTX-loaded micelles (PTX–NHOA) based on an *N*-acetylcysteine-modified hyaluronic acid–octadecylamine conjugate. Improved oral paclitaxel delivery through the intestinal mucus layer was achieved. Reprinted with permission from [155] (Copyright 2019 Elsevier Editorial).

Hyaluronic acid (HA) has also been utilized in some works as a vehicle for taxanes; for instance, Zhang et al. designed nanoparticles based on the conjugation of HA with octadecylamine (OA) [155]. The resulting complex was then functionalized with NAC with the objective of improving oral PTX delivery. In this way, these NPs were tested and compared with PTX-loaded nonfunctionalized HA–OA NPs, and also with Taxol^®^. Encapsulation efficiency was demonstrated to be optimal (~93%) for the first ones, while they also showed higher cellular uptake by endocytosis and better pharmacokinetic profile than the rest [155]. Therefore, the NAC functionalization was proven to be an effective permeation enhancer for drugs administered orally, since its thiol groups allow the NPs to exert considerably better adhesiveness to the intestinal mucous surface. Figure 12 shows a schematic illustration of the procedure followed in this study.

Apart from these two major polysaccharides, other interesting approaches have also been made. For example, Föger and coworkers carried out some in vivo investigations to evaluate a novel oral formulation of PTX based on thiolated polycarbophil [156]. Polycarbophil is the USP/NF compendial name of a family of polyacrylic acids loosely crosslinked with divinyl glycol with broad applications in biomedical and pharmaceutical fields [157], including as a laxative to treat constipation. When thiolated, it was demonstrated that P-gp inhibition was enhanced significantly, thus reducing tumor growth in mammary-cancer-induced rats more efficiently than intravenous paclitaxel [156]. Thiolated oligo(*p*-phenylenevinylene) (OPV–SH) has also been tested as a potential carrier for PTX into cancer cells by Zhou and coworkers [158]. In that case, a PTX–OPV conjugate was synthesized with the intention of reversing drug resistance by facilitating PTX delivery into the cells. OPV was chosen because of its biocompatibility and low cytotoxicity to normal cells. This work focused on taking advantage of the abundance of reactive oxygen species (ROS) inside the tumor cells to motivate crosslinking of the conjugate once it arrived at such cells. This mechanism is what they called “chemical locking”, and it was proven to successfully block tumor growth in xenotransplanted mice [158].

Levit et al. also recently demonstrated that NPs consisting of biodegradable poly(amino acid) and poly(2-deoxy-2-methacrylamido-d-glucose) (PMAG) can function as a reliable delivery system for paclitaxel in glutathione-rich conditions [159]. Great results were achieved when evaluated in A549 (human lung carcinoma cells) and MCF-7 (human breast adenocarcinoma) cells. Furthermore, thiolated sodium alginate has also shown to be promising, as proved by Chiu et al. [160]. This group synthesized disulfide-crosslinked NPs from the aforementioned material, and then coated them with fluorescein-labelled wheat germ agglutinin. The resulting NPs exerted favorable results for the delivery of docetaxel to human colon cancer cells, thus introducing this new polymer to the broad range of suitable materials for NP-dependent anticancer therapy.

Finally, dextran has also been used as part of thiolated carriers for hydrophobic therapeutics; for instance, as part of a hydrogel resulting from conjugation between thiolated human serum albumin and dextran. This albumin-based dextran conjugate demonstrated a broad range of potential biomedical applications as an efficient drug-delivery system [161].

Some studies with thiomers that focus on the delivery of other APIs apart from taxanes have also been published. These approaches can be considered as new horizons and references when tackling new formulations for the administration of taxanes. For instance, thiolated chitosan copolymer (functionalized with NAC) was utilized to coat curcumin-loaded nanostructured lipid carriers (NLCs) in an investigation carried out by Liu et al. The introduction of this thiol-rich coating allowed this formulation to exert better properties than the uncoated homologues, such as significantly enhanced transcorneal penetration and more sustained release of the loaded compound [125]. These advantages prove that thiomers may not only be useful for oral administration, but also for other approaches such as ophthalmic drug application. In fact, some controlled clinical trials have been performed to evaluate the benefits of thiolated polymers when used for ocular pathologies, such as those designed by Schmidl et al. [162]. They aimed to study the effect of CTS–NAC eye drops on tear film thickness in patients with dry eye syndrome. Results showed that the corneal status significantly improved in more than 60% of patients, with most of them starting to show beneficial results as early as 10 min after a single instillation of CTS–NAC [162]. This polymer has also been tested, along with CTS–NAP (CTS–*N*-acetylpenicillamine), as a platform to deliver doxorubicin (DOX) and antisense oligonucleotides (ASOND) targeting EGFR, which is a transmembrane receptor overexpressed in most of the cases of breast cancer with poor prognosis [114]. When evaluated on T47D cells (human breast epithelial tumor cells), these NPs showed similar properties as the ones stated before, such as sustained release, good encapsulation efficiency, and stability (especially those carrying ASOND), so the clinical potential of this formulation is obvious.

Continuing, thiolated hexanoyl glycol CTS (SH–HG–CTS) has also been investigated in the biomedical area as a plausible drug-delivery system. From the studies with such material, that of Cho et al. can be highlighted: this group synthesized SH–HG–CTS by first chemically modifying glycol CTS with hexanoic anhydride, and then introducing thiol groups by adding activated 3-mercaptopropionic acid [113]. SH–HG–CTS was demonstrated to have optimal thermo-gelling, mucoadhesive, and cytotoxic profiles, so further research should be conducted in order to possibly include this novel material in drug-delivery formulations.

Other works explore more complex formulations for NPs, such as the one developed by Sun and coworkers. These investigators prepared a nanosystem for the codelivery of DOX and plasmid encoding IL-12 gene (pIL-12), so that it could be used as a combination therapeutic method for metastatic breast cancer [136]. IL-12 has been reported to function as a cytokine that promotes antitumor activity, with immunomodulating, antiangiogenic, and antimetastatic actions that facilitate the suppression of tumor growth. The approach tackled by this group was based on DOX + IL-12-loaded polymetformin NPs, which were later completed with the addition of thiolated hyaluronic acid for better properties. These so-called micelleplexes exerted prolonged circulation in blood, efficient accumulation in tumors, excellent pIL-12 transfection, and sustained DOX release [136], so they hold great promise for ameliorating current chemo–gene combination therapies.

It is also interesting to mention that Bayat et al. explored how the functionalization of thiolated nanoparticles can affect drug delivery for childhood acute lymphoblastic leukemia (ALL), a study that had not been conducted before, even though ALL is the most common infant malignancy [163]. So far, most of the investigations regarding the use of nanoparticles for anticancer therapy have focused on treating solid tumors, so the fact that this group has developed an approach for hematological cancer is, by itself, worth mentioning. In this case, Bayat and coworkers demonstrate that NPs based on star polymers made out of poly(oligoethylene glycol methyl ether acrylate) (POEGA), or POEGA and poly(diethylene glycol ethyl ether acrylate) (PDEGA), can be efficiently used for the objective previously stated, with the latter being more successful [163]. These star polymers were also thiolated and functionalized with several very short ethoxy repeating units in the outermost part.

Overall, it was demonstrated that thiolated and functionalized star polymers can be used to safely improve cellular association and uptake of diverse APIs in childhood ALL, opening a broad range of new opportunities for boosting thiomers’ relevance in the treatment of blood cancers. In fact, some variants of these materials have already been developed for the delivery of siRNA and other chemical compounds involved in anticancer therapy (such as doxorubicin), as well as for magnetic resonance imaging contrast agents [163].

A brief list of selected studies regarding efficient taxane and other APIs delivery in cancer therapy (conducted with thiomer-based formulations) is summarized in Table 9. The results presented herein show the advantages of thiomers, such as those based on CTS and HA, in the effective and selective delivery of anticancer drugs to cells in solid tumors. In fact, these agents have become a pertinent tool in cancer care for the treatment of both nonadvanced disease and late-stage disease [164]. Nevertheless, more investigation is still needed, especially that regarding the testing of these novel formulation in human beings. They have been demonstrated to be potentially useful in the clinical field, not only in the delivery of chemotherapeutics through the gastrointestinal tract, but also in the oral administration of other APIs such as insulin [165,166]; even in this situation, most of the research work done so far could only be included in what we called the preclinical phase.

### 5.2. Thiomer-Based Conjugates for siRNA Administration

Interfering RNAs (iRNAs) are RNA molecules of 20–25 nucleotides of length whose structure makes them able to participate in a biological route known as ribo-interference or RNA-mediated interference [168]. Several studies have shown that this ancient process (dating evolutionarily even before the divergence of plants and animals [169]) can be considered as the oldest and most ubiquitous antiviral system [169]. In this way, several types of iRNAs have been investigated, but we will focus on small interfering RNAs (siRNA).

**Figure 13:**
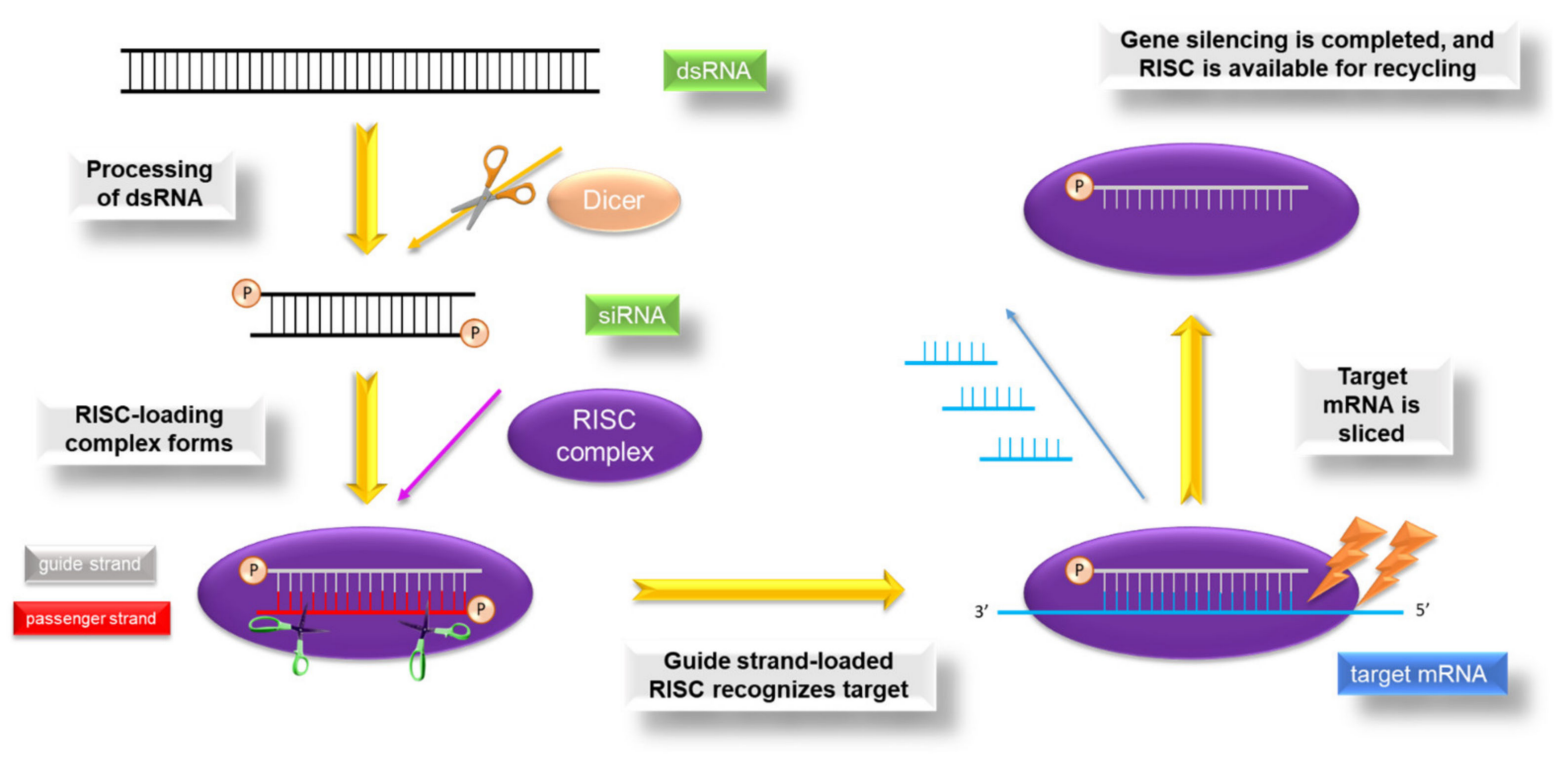
Basic functioning of RISC-based gene-silencing system.

The main goal for which cells use these particles is post-transcriptional gene silencing or, more particularly, gene downregulation, by targeting and destroying its messenger RNA (mRNA). In fact, the discovery of this cellular mechanism was so relevant that it made investigators Mello and Fire Nobel laureates in Medicine or Physiology in 2006. Structurally, siRNAs are double-stranded molecules that are synthesized by the cell in such a way that the sequence of one of its strands is complementary to that of the target messenger RNA. Actually, the first biological product that appears in the cell cytoplasm is a longer double-stranded piece of iRNA that is later cut into proper siRNAs by an enzyme called Dicer [170], as shown in Figure 13. Afterward, these fragments are captured by another enzyme, RNA-induced silencing complex (RISC), which will separate both strands and degrade the passenger strand; that is, the one with the same sequence as the target mRNA [170]. By doing this, RISC will incorporate the remaining strands (the antisense one) as if it was a cargo, and use it as a guide to identify the target mRNA via sequence complementarity [170]. Finally, once the correct mRNAs are recognized, RISC is able to cleave them, which lowers the amount of material that can be traduced, thus reducing the expression of the corresponding gene.

The clinical potential shown by this natural gene-suppression technique is enormous, giving that it occurs naturally during cell maturation, and is very selective and efficient [170]. In fact, as of 2013, around 22 iRNA-based therapeutic formulations were being investigated regarding the treatment of hereditary pathologies, viral infections, cancer, and other diseases that lack drug-based therapeutic options so far [170]. Moreover, it has been proved to be a very useful tool not only in the clinical field, but also in the biomedical and preclinical ones, since it allows the replication of pathologies in which genes are poorly expressed (knock-down animal models).

Nowadays, after discovering the possible usefulness of siRNAs in human therapy, investigations of delivery techniques of such molecules have boomed; namely, consideration of how fragile they are. For the iRNA machinery to function, siRNA molecules must be efficiently transported to the interior of the target cells while being protected from enzyme degradation and other damaging processes at the same time (especially when administered orally) [140,170].

Currently, there are some synthetic, nonviral delivery agents for siRNA that are on the rise for showing to be the most promising, among which mainly lipid-based formulations are found [140]. Some cationic molecules (including several polymers) have been used in successfully transporting siRNA in mammal cells while also avoiding major toxic effects [140]. In any case, direct conjugation of the delivery agents has been proven to facilitate both selectivity and efficacy in the delivery process, and also reduce the potential immune stimulation against the double-stranded RNAs (which is undesirable). As an example of the first aspect, a study conducted by Soutschek et al. demonstrated that cholesterol-bound siRNA resulted in the efficient silencing of the apoB mRNA specifically in liver and jejunum cells [140,171]. Moving on to the second idea, it has been suggested that siRNA can stimulate TLR receptors in dendritic cells if endocytosed and processed into their constituent oligonucleotides [172], so it poses a risk to the patient if their immune system were to react violently against it (some off-target gene effects can be associated with this inflammatory response). With this idea in mind, some groups explored the option of incorporating 2’-*O*-methyl-modified uridine or guanosine residues into the interfering RNAs to avoid that immune stimulation, and some promising results were obtained [173].

Having introduced the main aspects of the status quo of siRNA in the clinical area, the attention will now be focused on thiolated polymers as an efficient delivery agent. J.Y. Yhee et al. studied the formation of intravenous-delivered NPs from self-crosslinked thiolated glycol chitosan as a way to effectively transport siRNA to cancer cells [124]. The main purpose of using this technique was to make tumor cells more sensitive to chemical chemotherapeutics by blocking the efflux pump [124]. Therefore, the administration of these NPs is intended to accompany dosages of other therapeutic elements: the siRNAs is targeted against P-glycoprotein mRNA, which acts as an efflux pump in cancer cells and participates in the clearance of the administered chemical compounds. Moreover, the selectivity of NPs uptake in cancer cells is improved naturally by the EPR effect [124].

With this in mind, if the expression of this kind of protein is downregulated, tumor cells will be indirectly made less resistant to proper drugs; that is, overcoming drug resistance in cancer [124]. That is why this is thought to be a possible adjuvant or supplementary therapy to what already has been achieved. Some of the obvious benefits of this medication would be that subtherapeutical doses of chemotherapeutics could now be efficient against certain tumors [124]; if the dosage is reduced, so will the systemic effects associated with the toxicity of the compound. So, even though it is not thought to be a monotherapeutic treatment, it is a promising tool in the oncology field.

As stated previously, CTS was functionalized with thiol groups; nonetheless, it was not the only modification that has been conducted. It has been demonstrated that certain modifications of siRNA could make it more avid to binding to its corresponding carrier, thus improving the stability of the formulation [124]. In particular, reducible poly-siRNA has been proposed as a possible solution for low delivery efficiency of these molecules [100,174]. The formation of this poly-siRNA would be plausible via thiolation of the 5′-end of the two strands of each RNA fragment, which would allow these double-stranded RNA molecules to not only conjugate among themselves, but also with the thiolated polymer, thus forming an extensive network of interconnected disulfide links that could easily be cleaved in a reductive ambiance, like the one found in the cellular interior [100,122,124,174]. This glutathione-rich compartment would allow disulfide links to break, hence disintegrating NPs once captured by the target cells and efficiently delivering monomeric siRNA to the corresponding machinery in cellular cytosol [100]. Moreover, if cationic thiolated polymers are used, such as CTS-based thiomers, electrostatic interactions are also utilized: the NP network is further strengthened because of the net negative charge exhibited by poly-siRNA, so it can be successfully incorporated into carriers with cationic charge density, such as thiolated CTS derivatives [124].

**Figure 14:**
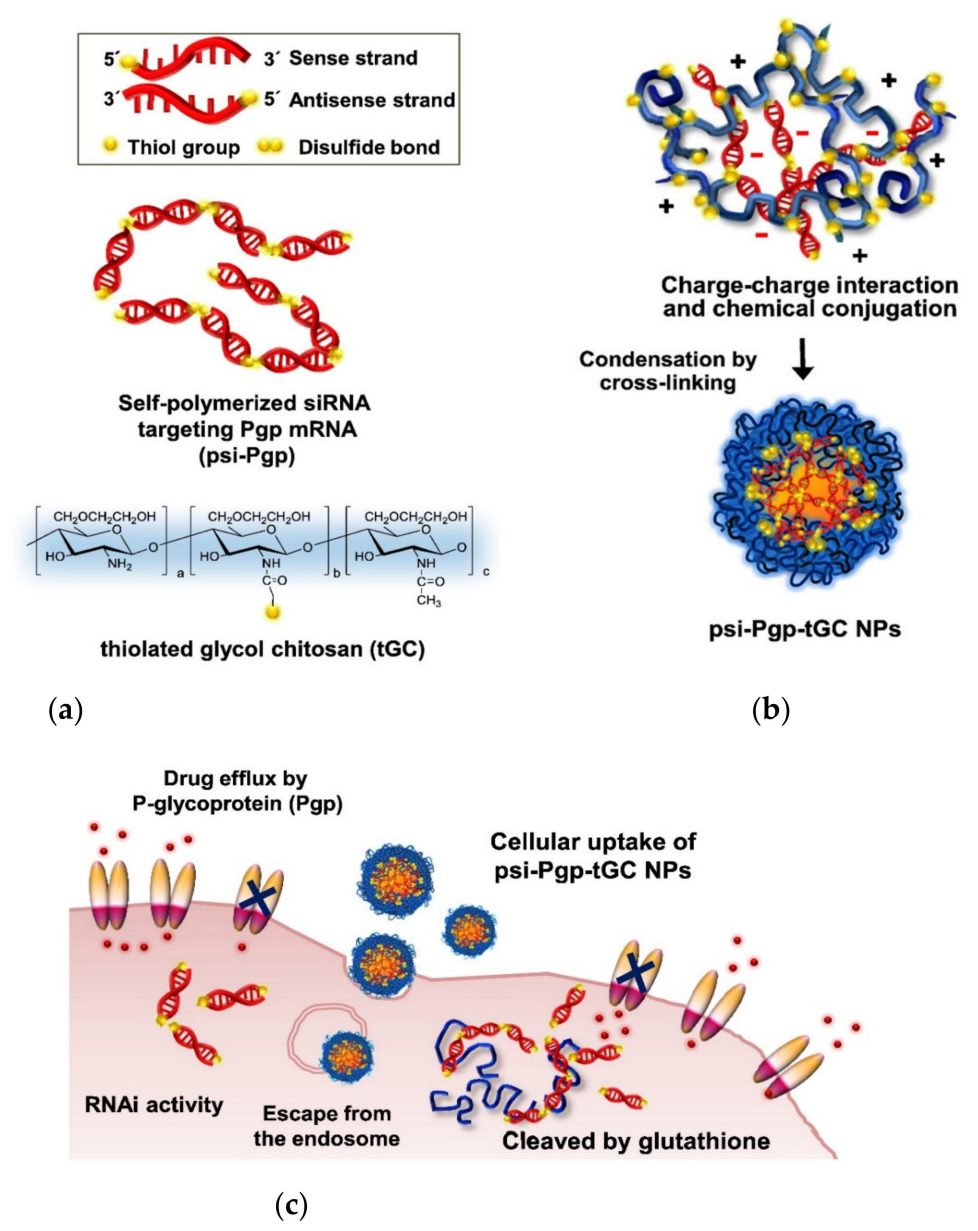
Formation, intracellular delivery, and mechanism of action of thiolated glycol chitosan–nanoparticles containing siRNA against P-gp. (**a**) Thiolation of siRNA and glycol chitosan. (**b**) Formation of NPs. (**c**) Glutathione-dependent siRNA delivery in cells. Reprinted with permission from [124] (Copyright 2015 Elsevier Editorial).

For example, thiolated glycol chitosan–poly-siRNA NPs were shown to be a useful tool to prevent enzyme degradation of the ribonucleic acids (if injected into the bloodstream alone, its half-life was significantly shortened) and adequately release the molecules when the conditions favored the cleavage of the disulfide bridges [124]. Furthermore, the system exhibited excellent stability in serum, a better pharmacological profile than the chemical adjuvant alone, and no unintended immune stimulation. In conclusion, the results showed that the P-gp downregulation was quantitatively achieved (–62% of P-gp expression in cancer cells [124]), which consequently led to a greater concentration of the adjuvant chemotherapeutic inside the tumor cells. Figure 14 represents a schematic illustration of the procedure followed in this study.

The synthesis of NPs based on thiolated glycol chitosan and poly-siRNA was an approach also tackled by Lee et al. in 2011. Here, they specified that NPs were able to form thanks to simultaneous self-crosslinking and charge–charge interactions [122]. Eventually, these investigators came to similar conclusions as those presented previously: this nanostructure allowed tumor-targeted mono-siRNA release, which could be taken by cells efficiently for optimal gene silencing. These experiments were carried out in vivo by injecting the preparation into tumor-bearing mice, and the targeted gene was that expressing the vascular endothelial growth factor (VEGF). Consequently, results showed that angiogenesis in tumors treated with small amounts of siRNA was significantly reduced, leading to a quite successful conclusion [122].

It is evident that chemo–siRNA combination therapy shall be considered as a future option to significantly lower tumor growth rates while avoiding major signs of excessive systemic toxicity; in fact, this is an investigational approach already in the making for cases such as triple-negative breast cancer [175].

Similar studies have been conducted by other groups that intended to exploit the potential of siRNA, such as that of Work and coworkers [176]. This specific example explored a novel formulation for the delivery of siRNA based on an *N*-(2-hydroxypropyl)methacrylamide-*s*-*N*-(3-aminopropyl)methacrylamide (HPMAm-*s*-AMPAm) copolymer, which was synthesized via RAFT-polymerization [176]. HPMAm was the main component (90 mol %) of the copolymer described by the authors, because it tends not to be immunogenic and it is soluble in water-based solvents [176]. In a similar fashion to what already has been described, this approach permitted satisfactory poly-siRNA cleavage in glutathione-rich conditions. In this case, incorporation of folate into the formulation was also attempted to achieve targeting of the system toward cancer cells, as it is a natural moiety that is able to target these cells [176]. By doing that, enhanced specificity regarding the site of action of the NPs was expected [177]. In this case, no thiolated polymer was used, but it is an approach that can easily serve as the basis for the synthesis of new HPMAm-*s*-AMPAm-based thiomers.

Moreover, the strategy tackled by Varkouhi et al. is also worth mentioning. It was based on the synthesis of another nonviral vector for nucleic acid delivery, in this case in the form of polyplexes from thiolated *N*,*N*,*N*-trimethylated chitosan (thiolated TMC) [178]. By generating this derivative of CTS, the investigators managed to improve safety, effectiveness, and solubility in aqueous solvents at neutral pH. Several trials were carried out comparing thiolated TMC to its unthiolated homologue, and also to the lipidic transfection agent Lipofectamine. In most of the experiment, the thiolated TMC demonstrated to be markedly better regarding gene-silencing activity, protection against degrading nucleases, release kinetics, cellular uptake, and overall clinical profile [178].

Additionally, this group studied the influence of HA as both a prevalent macromolecule in the extracellular fluids and as part of the copolymer forming the NPs. In this sense, there is evidence that supports the fact that hyaluronic acid can be considered as a double-edged sword. On the one hand, the gene-silencing efficiency of these polyplexes has been shown to be significantly reduced in the presence of hyaluronic acid, and this is something to take into account, since this glycosaminoglycan is rather abundant in our connective, epithelial, and neural tissue. For instance, this negative connotation was evaluated in a study conducted by Van de Wetering et al., in which they figured out that transfection efficiency can be diminished up to fourfold [179]. Therefore, it is not surprising that the study inferred that hyaluronic acid may act as a competitor molecule with the NPs. Apart from this, serum proteins might also play an important role in lowering the cellular uptake of NPs [178].

On the other hand, if incorporated into the NPs themselves, HA has demonstrated to add stability to the overall structure of the NPs; Varkouhi et al. tested this hypothesis by synthesizing NPs from both thiolated and nonthiolated TMC in combination with either thiolated or nonthiolated HA [178]. From the four resulting conjugates, the one that showed the highest gene-suppression activity was the mixture of thiolated TMC and thiolated HA, probably because the stability of the polyplex was enhanced thanks to the formation of more disulfide bonds between both types of polymers [178]. Furthermore, other works used thiolated HA in a similar fashion to what was presented in the section on taxanes; that is, as a detachable shell covering a more consistent core. An example of this was developed by Yin and coworkers using octyl-modified polyethyleneimine containing disulfide linkages (PSR) as the core of the NPs [180]. In this case, the thiolated HA covering not only served as protection for the drug-loaded cores, but it also allowed a more selective tumor-targeting thanks to the fact that HA is a molecule recognized by cluster of differentiation 44 (CD44), which is usually overexpressed in some neoplasms. This system was proven to effectively be able to codeliver hydrophobic chemotherapeutics and hydrophilic siRNA, maximizing synergistic antitumor efficacy via a GSH-mediated release of both components [180].

Another example involving thiolated HA is that presented by Zhou et al. In this case, cross-linked thiolated HA was used to form an anionic outer shell to protect, stabilize, and optimize the siRNA-loaded core. This core was not designed the same way as the previous example; instead, it was synthesized so that the siRNA would be capable of forming the core without using other polymers. These investigators managed to do this by stabilizing siRNA with calcium phosphate as a core inside the thiolated HA shell [31]. The consequent studies with intravenous injection of the formulation demonstrated excellent transfection efficiency, selective release of siRNA into the cytosol (via GSH-dependent dismantling and successful endosomal escape), no apparent toxicity, and also around 80% gene-suppressing efficacy in vitro for both luciferase and B-cell lymphoma 2 (Bcl-2) genes [31]. Bcl-2 is part of a family of regulator proteins that modulate cell death; in particular, it plays a relevant role in inhibiting the actions of proapoptotic proteins (thus allowing cells to survive). That is why its silencing in cancer cells will lower tumor growth, because it will be promoting apoptosis of those cells.

**Figure 15:**
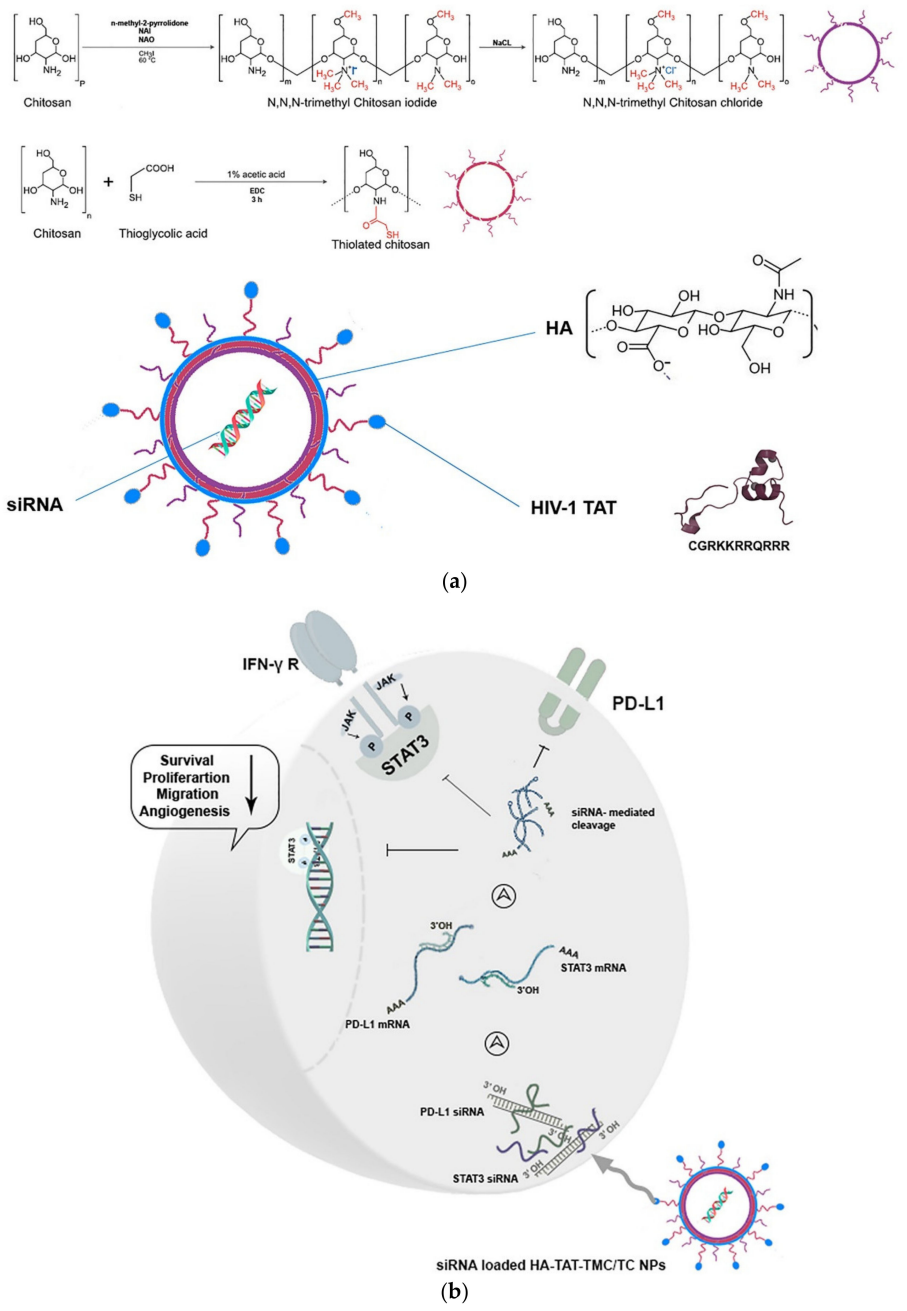
(**a**) Formation of siRNA-loaded TAT–HA–TMC–TC NPs. (**b**) Intracellular delivery and mechanism of action of siRNA-loaded TAT–HA–TMC–TC NPs. Reprinted with permission from [181] (Copyright 2021 Elsevier Editorial).

Three more studies can be highlighted as relevant examples of successful delivery systems for siRNA. The first one was thought to be an immunotherapy method to boost the natural ability of the human immune system to destroy cancer cells. In fact, similar approaches (but using antibodies instead of siRNA) tackled by Allison and Honjo were so revolutionary and had so much impact that they resulted in the concession of the 2018 Nobel Prize in Physiology and Medicine to the abovementioned investigators. As immunotherapeutic agents are on the rise, Bastaki et al. contributed to the field with their experiments in the delivery of siRNAs to silence programmed cell death-ligand 1 (PD-L1) and signal transducer and activator of transcription-3 (STAT-3) genes [181]. By doing so, they designed TMC and thiolated chitosan NPs to hold the desired siRNA, as shown in Figure 15. Moreover, the NPs were completed by conjugation with HIV-1-derived TAT peptide and HA. The in vivo experiments shed light on promising results regarding significant downregulation of PD-L1 and STAT-3 genes. Consequently, important restriction of tumor growth was achieved: proliferation, migration, and angiogenesis of breast and melanoma cancer cells lines were strikingly lowered [181].

Second, in 2014 Muthiah and coworkers registered an NP-based formulation for efficient anticancer gene therapy via silencing of serine-threonine protein kinase Akt1′s gene. This protein has been reported to participate in biochemical pathways participating in the suppression of apoptosis, so the hypothesis suggested by this investigation group is that inhibiting its action should promote an antiproliferative response. In this case, polyethylenimine (PEI) was thiolated in order to synthesize the NPs responsible for the delivery of also-thiolated siRNA [182]. Disulfide bonds between the polymer and the siRNA ensured stability of the formulation. The antitumor effects of these NPs were tested using mouse colon cancer cells and in vivo mouse tumor models, in which it was proven that cellular uptake was enhanced and neoplasm proliferation was reduced via effective downregulation of Akt1 [182].

Last but not least, thiolated gelatin has been used for the same purpose as well [183]. Thus, siRNA gelatin NPs were administered to tumor-bearing mice to evaluate the pharmacological effects of this approach in vivo. The authors demonstrated that thiolated gelatin effectively protects siRNA molecules from degradation while simultaneously allowing their delivery to target cells in reductive conditions. It was concluded that these NPs can motivate significant gene silencing in vivo without remarkable toxicity [183].

A brief list of selected studies regarding efficient anticancer siRNA delivery (conducted with thiomer-based formulations) is displayed in Table 10. Summarizing everything stated, it is evident that these novel siRNA-based strategies have great potential for cancer treatments. They may be used as adjuvant therapy to the administration of chemotherapeutics in order to reduce the doses needed, and thus the associated toxicity. More clinical trials are needed to ensure the safety and effectiveness of these, and therefore to make possible their commercialization and usage in lieu of other more traditional and harsh therapeutic treatments. Thiomers have demonstrated to be an optimal delivery system for siRNA, but again, more studies need to be conducted to enable their commercialization as safe and effective antitumor formulations.

## 6. Conclusions

In summary, attractive and reliable methods are available for preparing NPs from CTS- and HA-based thiomers with smart properties for their applications in cancer therapy, particularly NP formulations with taxanes and siRNA. The most interesting and reliable synthetic methods available for preparing CTS- and HA-based thiomers have been highlighted, with their advantages and drawbacks. The reported procedures could be extended to other relevant natural or synthetic polyelectrolytes, with a consequent potential boost in this research field. We anticipate that this work could inspire the development of cutting-edge thiomer-based NPs with improved clear advantages in drug delivery for cancer therapies, and with other therapeutical uses.

## Figures and Tables

**Table 3:** Disulfides used for the formation of preactivated chitosan-based thiomers by 1-ethyl-3-(3-dimethylaminopropyl)carbodiimide (EDAC)-mediated coupling reactions (Reaction 2 in Figure 8) between the carboxylic acid moiety from the reagent and the amino groups from the polymer.

Reaction Code	Compound		Ref.	Reaction Code	Compound		Ref.
	Formula	Abbreviations			Formula	Abbreviations	
**2**	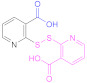 2,2’-Dithiodinicotinic acid	2,2′-DTNA	[72]	**2**	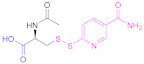 *N*-acetyl-*S*-((5-carbamoylpyridin-2-yl)thio)-l-cysteine	NAC-6-MNAm	[120]

**Table 4:** (Protected) thiol-containing active esters (*N*-hydroxysuccinimide esters) for thiolation of multiamino polymers and chemical structure of the reactive acetimidate isopropyl (acetylthio)acetimidate (*i*-PATAI). Reagents used for Reactions 3 and 4 in Figure 8.

Reaction Code	Compound		Ref.	Reaction Code	Compound		Ref.
	Formula	Abbrev.			Formula	Abbrev.	
**3**	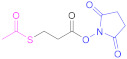 *N*-Succinimidyl-*S*-acetylthiopropionate	SATP	[60]	**3**	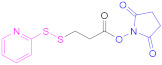 *N*-Succinimidyl 3-(2-pyridyldithio) propionate	SPDP	[55,93]
**3**	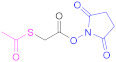 *N*-Succinimidyl *S*-(acetyl) thioglycolate	SATA	[80,123]	**3**	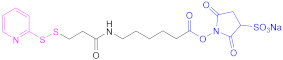 *N*-Sulfosuccinimidyl 6-(3-(pyridin-2-yldisulfaneyl) propionamido) hexanoate	Sulfo-LC-SPDP	[122,124]
**3**	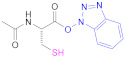 NAC activated	--	[125]	**3**	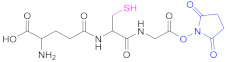 GSH activated	--	[60]
**4**	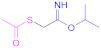 Isopropyl (acetylthio)acetimidate	*i*-PATAI	[95,121]				

**Table 5:** Nucleophilic thiolating agents used for nucleophilic substitution reactions of multiamino polymers (Reactions 5 and 6 in Figure 8).

Reaction Code	Compound		Ref.	Reaction Code	Compound		Ref.
	Formula	Abbreviations			Formula	Abbreviations	
**5** **6**	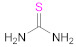 Thiourea	--	Reacc. 5: [51,52,54] Reacc. 6: [49,52]	**6**	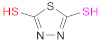 2,5-Dimercapto-1,3,4-thiadiazole	DMTD	[50]
**6**	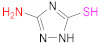 3-Amino-1,2,4-triazole-5-thiol	AZ	[127]				

**Table 8:** NPs formation from hyaluronic acid and chitosan-based thiomers.

System	Particle Formation Method	Crosslinker(s)	APIs	Thiomer	NP Size (nm)/PdI	Ref.
Microparticles	Spray-drying technique	---	FDA	CTS–NAC CTS–GSH	800–2270/---	[110]
NP	Self-assembly of the amphiphilic material	---	Docetaxel	CTS–GSH–*graft*–PMMA	<200/<0.15	[27]
NP	Self-assembly of the amphiphilic material	Air core XrL (ultrasonication)	Methotrexate	CM–CTS–Ph–SH ^a^	160/0.055	[25]
NP	Ionic gelation	Na_2_SO_4_	DOX or ASOND	CTS–NAC CTS–NAP	150–300/---	[114]
NP	Ionic gelation	TPP Ca^2+^	FD4	CTS–TGA with TPP PAA–Cys with Ca^2+^	220–280/---	[29]
NLC	Polyelectrolyte complex formation	Negatively charged CUR-loaded NLC	Curcumin	CTS–NAC	89/---	[125]
Hybrid nanoplex	Polyelectrolyte complex formation	HA of reduced Mw	FD4	QA–CTS–TGA–SH or QA–CTS–TGA–SS–6-MNAm	371–376/0.39–0.41	[116]
Micelleplexes	Double polyelectrolyte complex formation: 1. pIL-12 + DOX-Loaded polymetformin micelles 2. Micelleplexes + HA-SH	For step 1: pIL-12 For step 2: Polymetformin-based micelleplexes	DOX and pIL-12	HA–SH	98–192/---	[136]
Hybrid nanoplex	Polyelectrolyte complex formation and ionic gelation	Negatively charged insulin TPP	Insulin	Thiomalyl–CTS	365/---	[24]
Nanosuspension	Polyelectrolyte complex formation and ionic gelation	HA TPP	Isoniazid	CTS–TGA	300–500/---	[23]
NP	Polyelectrolyte complex method and disulfide bond formation	bFGF Thiol oxidation (air, pH 7.4)	bFGF	Carboxymethyl(sulfate)–CTS–thiobutylamidine	218–290/---	[30]
NP	Polyelectrolyte complex method and disulfide bond formation	Poly-siRNA Thiol oxidation (air, pH 8.0, 37 °C)	siRNA	Glycol–CTS–SH	~300/---	[122,124]
NP	Polyelectrolyte complex method, Ionic gelation and disulfide bond formation	Poly-siRNA Calcium phosphate	siRNA	HA–SH	169/0.108	[31]

^a^ Thiolating agent: 4-mercaptobenzoic acid. 6-MNAm: 6-Mercaptonicotinamide; ASOND: antisense oligonucleotide; bFGF: basic fibroblast growth factors; CM: carboxymethyl; CTS: chitosan; CUR: curcumin; Cys: cysteine; DOX: doxorubicin; FD4: fluorescein isothiocyanate–dextran; FDA: fluorescein diacetate; GSH: glutathione; HA: hyaluronic acid; HPMAm: hydroxypropylmethacrylamide mono- or dilactate; Mw: molecular weight; NAC: *N*-acetylcysteine; NAP: *N*-acetylpenicillamine; NLC: nanostructured lipid carrier; PAA: poly(acrylic acid); pIL-12: plasmid-encoding IL-12 gene; PdI: polydispersity index: PMMA: poly(methyl methacrylate); QA: quaternary ammonium; siRNA: small interfering RNA; TGA: thioglycolic acid; TPP: tripolyphosphate; XrL: crosslinked.

**Table 9:** Summary of selected studies regarding efficient delivery of taxanes and other APIs in cancer therapy, conducted with thiomer-based formulations.

Thiomer	Formulation	Loaded Compound	Cancer Cells Studied In Vitro	Ref.
Thiolated CTS	Nanoparticles (PMMA core + CTS–GSH conjugate coating)	Paclitaxel	NIH 3T3 and T47D (breast carcinoma) cells	[37]
Thiolated CTS	Nanoparticles (PMMA core + thiolated chitosan coating)	Docetaxel	Caco-2 (colorectal adenocarcinoma) and MCF-7 (breast carcinoma) cell lines	[27]
Thiolated CTS	Nanoparticles (PLA–PCL–TPGS core + thiolated chitosan coating)	Paclitaxel	A549 (adenocarcinomic human alveolar basal epithelia) cells	[151]
Thiolated HA–OA	Nanoparticles (HA–OA conjugate functionalized with NAC)	Paclitaxel	Caco-2 cells and Caco-2/HT29 cell monolayer (human colon cancer cell lines)	[155]
Thiolated polycarbophil	Nanoparticles	Paclitaxel	Only in vivo studies (wild-type rats vs. mammary-cancer-induced rats)	[156]
Thiolated OPV	Nanoparticles	Paclitaxel	A549 (adenocarcinomic human alveolar basal epithelia) cells	[158]
Thiolated sodium alginate	Nanoparticles (fluorescein-labelled wheat germ agglutinin-conjugated disulfide-crosslinked sodium alginate)	Docetaxel	HT-29 (human colon cancer) cells and L929 (mouse fibroblast) cells	[160]
Thiolated PEG + maleimide-functionalized HA	Microbeads	Docetaxel	NIH/3T3 (mouse embryonic fibroblast) cells and 4T1 (mouse breast cancer) cells	[167]
Dextran ^a^	Hydrogel (dextran + thiolated human serum albumin)	Paclitaxel	-	[161]
PMAG + poly(amino acid)	Nanoparticles	Paclitaxel	A549 (human lung carcinoma) and MCF-7 (human breast adenocarcinoma) cells	[159]
Thiolated CTS ^c^	Nanostructured lipid carrier (lipid core + thiolated chitosan coating)	Curcumin	Isolated rabbit cornea cells	[125]
Thiolated CTS ^c^	Nanoparticles (based on CTS–NAC or CTS–NAP)	Doxorubicin and antisense oligonucleotides	T47D (human breast epithelial tumor) cells	[114]
Thiolated hexanoyl glycol CTS ^c^	Mucoadhesive thermogelling polymer	-	HeLa (cervical cancer) cells, human fibroblasts, epithelial cells, and epithelial cell aggregate	[113]
Thiolated HA ^c^	Micelleplexes (polymetformin + hyaluronic acid)	Doxorubicin and plasmid-encoding IL-12 gene	4T1 (mouse breast cancer) cell line	[136]
Thiolated amidized glycol CTS ^c^	Dual secured nano-sting	Melittin and other amphipathic peptides	MCF-7 (breast carcinoma), HCT-116 (human colon cancer), SKOV-3 (human ovarian cancer), and NCI/ADR–RES (ovarian tumor) cell lines	[55]
PLGA and OCTS ^b,c^	Nanoparticles	CHC and CTX	U251 and SW1088 (glioma cell lines)	[80]
Thiolated carboxymethyl chitosan ^c^	Nanoparticles	Methotrexate	HeLa (cervical cancer) cells	[25]
POEGA + PDEGA (star polymers)	Nanoparticles	-	Jurkat (T-ALL) and Nalm-6 (B-ALL) cancer cell lines	[163]

^a^ This work did not use a thiolated polymer per se for the synthesis of nanoparticles, but involved a polymer linked with disulfide bonds to thiolated human serum albumin. ^b^ This work did not use a thiolated polymer *per se* for the synthesis of nanoparticles but involved a PEG crosslinker to link the loaded compounds to the polymer via Michael reaction. ^c^ These works correspond to approaches that were not directly applied to taxanes, but can be considered as new horizons and references when tackling newer formulations for the administration of these APIs. CHC: alpha-cyano-4-hydroxycinnamic acid; CTS: chitosan; CTX: cetuximab; GSH: reduced glutathione; HA–OA: hyaluronic acid–octadecylamine; IL-12: interleukin 12; NAC: *N*-acetylcysteine; NAP: *N*-acetylpenicillamine; OCTS: oligomeric chitosan; OPV: oligo(*p*-phenylenevinylene); PDEGA: poly(diethylene glycol ethyl ether acrylate); PEG: polyethylenge glycol; PLA–PCL–TPGS: poly(lactide-*co*-*ε*-caprolactone)-d-*α*-tocopheryl polyethylene glycol 1000 succinate; PLGA: poly(lactic-*co*-glycolic acid); PMAG: poly(2-deoxy-2-methacrylamido-d-glucose); PMMA: poly(methyl methacrylate); POEGA: poly(oligoethylene glycol methyl ether acrylate).

**Table 10:** Summary of studies regarding efficient anticancer siRNA delivery conducted with thiomer-based formulations.

Thiomer	Formulation	Loaded Compound	Cancer Cells Studied In Vitro	Ref.
Thiolated glycol CTS	Nanoparticles	MDR-1 siRNA	MCF-7/ADR (Adriamycin-resistant breast cancer cells)	[124]
Thiolated glycol CTS	Nanoparticles	VEGF siRNA	RFP-expressing B16F10 (murine melanoma) cells. In vivo studies in SCC-7 and RFP-B16F10 tumor-bearing mic	[122]
HPMAm-*s*-AMPAm ^a^	Multiconjugates	siRNA and folate	-	[176]
Thiolated TMC (both alone and with HA)	Polyplexes	siRNA	H1299 (human lung cancer) cells	[178]
Thiolated HA + PSR	Nanoparticles (PSR core + thiolated hyaluronic acid coating)	Hydrophobic chemotherapeutics + siRNA	A549 (adenocarcinomic human alveolar basal epithelia) cells	[180]
Thiolated HA	Nanoparticles (siRNA with calcium phosphate core + thiolated hyaluronic acid coating)	Bcl-2 siRNA	B16-F10 (murine melanoma) cells	[31]
Thiolated chitosan + TMC	Nanoparticles (thiolated chitosan + TMC completed with HA and HIV-derived TAT peptide)	PD-L1 and STAT-3 siRNA	B16-F10 (murine melanoma) and 4T1 (mouse breast cancer) cell lines	[181]
Thiolated PEI	Nanoparticles	Akt1 siRNA	CT-26 (mouse colon cancer) cells. In vivo studies in mouse tumor models	[182]
Thiolated gelatin	Nanoparticles	siRNA	RFP-expressing B16F10 (murine melanoma) cells. In vivo studies in tumor-bearing mice	[183]

^a^ This work did not use a thiolated polymer for the generation of nanoparticles, but it is an approach that can easily serve as the basis for the synthesis of new HPMAm-*s*-AMPAm-based thiomers. Bcl-2: B-cell lymphoma 2 gene; CTS: chitosan; HA: hyaluronic acid; HPMAm-*s*-AMPAm: *N*-(2-hydroxypropyl)methacrylamide-s-*N*-(3-aminopropyl)methacrylamide; MDR: multidrug resistance gene; PD-L1: programmed cell death-ligand 1; PEI: polyethylenimine; PSR: octyl-modified polyethyleneimine containing disulfide linkages; siRNA: small interfering ribonucleic acid; STAT-3: signal transducer and activator of transcription-3; TMC: *N,N,N*-trimethylated chitosan; VEGF: vascular endothelial growth factor.

## Abbreviations

2,2′-DTNA	2,2′-Dithiodinicotinic acid
2-MNA	2-Mercaptonicotinic acid
4-MBA	4-Mercaptobenzoic acid
6,6′-DTNA	6,6′-Dithiodinicotinic acid
6,6′-DTNAm	6,6′-Dithiodinicotinic amide
6-MNA	6-Mercaptonicotinic acid
6-MNAm	6 Mercaptonicotinamide
APIs	Active pharmaceutical ingredients
ASOND	Anti-sense oligonucleotides
Bcl-2	B-cell lymphoma 2
CD44	Cluster of differentiation 44
CDI	1,1′ Carbonyldiimidazole
CSA	Cysteamine
CTS	Chitosan
CTX	Cetuximab
Cys	L-Cysteine
DCM	Dichloromethane
DDS	Drug delivery system
DIC	*N*,*N*′-Diisopropylcarbodiimide
DMAP	4-(Dimethylamino)pyridine
DMF	*N*,*N*-Dimethylformamide
DOX	Doxorubicin
DTNB	5,5′-Dithiobis-(2-nitrobenzoic acid)
DTPH	3,3′ Dithiobis(propanoic hydrazide)
DTT	Dithiothreitol
DTX	Docet-axel
ECM	Extracellular matrix
EDAC	1-Ethyl-3-(3-dimethylaminopropyl)carbodiimide
EGFR	Epidermal growth factor re-ceptor
EPR	Enhanced permeability and retention
FD4	Fluorescein isothiocyanate–dextran
FDA	Fluorescein diacetate
GSH	Reduced Glutathione
GSSG	Oxidized Glutathione
HA	Hyaluronic acid
HAase	Hyaluronidase
HOBt	N Hydroxybenzotriazole
HPMAM-s-AMPAM	N (2 Hydroxypropyl)methacrylamide-s-N-(3-aminopropyl)methacrylamide
HSA	Human serum albumin
i-PATAI	Isopropyl (acetylthio)acetimidate
iRNAs	Interfer-ing RNAs
LDC	Lipid drug conjugate
MBA	4-Mercaptobutanoic acid
MDR	Multi-drug resistance
MHA	6-Mercaptohexanoic acid
MOA	8-Mercaptooctanoic acid
MPA	Mercaptopropanoic acid
MPA	3-Mercaptopropanoic acid
MPPT	3-Methyl-1-phenylpyrazole-5-thiol
mRNA	Messenger RNA
MUA	11 Mercaptoundecanoic acid
NAC	N-Acetylcysteine
NAP	N-Acetylpenicillamine
NHS	N-hydroxysuccinimide
NLC	Nanostructured lipid carriers
NPs	Nanoparticles
OA	Octadecylamine
PAA	Poly(acrylic acid)
PD-L1	Programmed cell death-ligand 1
Pec	Pectin
PEG	Polyethyleneglycol
PEI	Polyethylenimine
P-gp	P-glycoprotein
pIL-12	Plasmid encoding IL-12 gene
PLA-PCL-TPGS	Poly(lactide-co-ε-caprolactone)-D-α-tocopheryl polyethylene glycol 1000 succinate
PLGA	Poly(lactic-co-glycolic) acid
PMAA	Poly(methacrylic acid)
PMMA	Poly(methyl methacrylate)
Poly-siRNA	polymeric siRNA
PTX	Paclitaxel
QA-CTS-TGA-SH	Quaternary ammonium CTS thiomers
RISC	RNA-induced si-lencing complex
ROS	Reactive oxygen species
SATA	N-succinimidyl-S-(acetyl) thi-oglycolate
siRNA	Small interfering RNA
SLN	Solid lipid nanoparticles
STAT-3	Signal transducer and activator of transcription-3
Sulfo-NHS	N-Hydroxysulfosuccinimide sodium salt
TCEP	Tris(2 carboxyethyl)phosphine
TGA	Thioglycolic acid
TJs	Tight junctions
TMC	*N*,*N*,*N*-trimethylated chitosan
TPP	Tripolyphosphate
VEGF	Vascular endothelial growth factor
XrL	Crosslinked
